# (μ-5-Carboxy­benzene-1,3-dicarboxyl­ato-κ^2^ *O* ^1^:*O* ^3^)bis­[bis­(2,2′-bipyridine-κ^2^ *N*,*N*′)copper(II)] 5-carboxy­benzene-1,3-dicarboxyl­ate 2,2′-bipyridine solvate trideca­hydrate

**DOI:** 10.1107/S1600536808001852

**Published:** 2008-01-23

**Authors:** Jaromír Marek, Zdeněk Trávníček

**Affiliations:** a Department of Inorganic Chemistry, Faculty of Science, Palacký University, Křížkovského 10, CZ-771 47 Olomouc, Czech Republic; b Laboratory of Functional Genomics and Proteomics, Institute of Experimental Biology, Faculty of Science, Masaryk University, Kamenice 5, CZ-625 00 Brno, Czech Republic

## Abstract

The asymmetric unit of the title complex, [Cu_2_(C_9_H_4_O_6_)(C_10_H_8_N_2_)_4_](C_9_H_4_O_6_)·C_10_H_8_N_2_·13H_2_O, comprises two formula units. The two Cu^II^ centres are bridged by a 5-carb­oxy­benzene-1,3-dicarboxyl­ate (Hbtc) ligand. Each of the metal centres is bonded to four N atoms of two bidentate 2,2′-bipyridine ligands (bpy) and one O atom of the Hbtc ligand in a highly distorted square-pyramidal geometry. The secondary structure is stabilized by a variety of O—H⋯O hydrogen bonds and π–π stacking inter­actions connecting the complex cations, Hbtc anions, bpy and water mol­ecules of crystallization. Three water molecules are disordered over two positions, with site occupancy factors of *ca* 0.8 and 0.2.

## Related literature

For related literature, see: Allen (2002 ▶); Pech & Pickardt (1988 ▶); Chui *et al.* (1999 ▶); Holmes *et al.* (2004 ▶); Wang *et al.* (2005 ▶); Cooper *et al.* (2005 ▶).
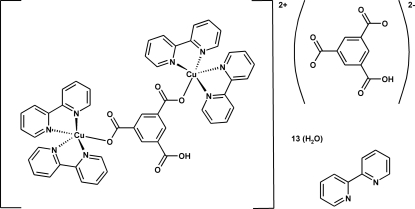


## Experimental

### 

#### Crystal data

[Cu_2_(C_9_H_4_O_6_)(C_10_H_8_N_2_)_4_](C_9_H_4_O_6_)·C_10_H_8_N_2_·13H_2_O
*M* *_r_* = 1558.45
Monoclinic, 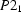

*a* = 14.4638 (2) Å
*b* = 19.7448 (2) Å
*c* = 25.0638 (3) Å
β = 99.2051 (14)°
*V* = 7065.66 (15) Å^3^
*Z* = 4
Mo *K*α radiation
μ = 0.69 mm^−1^
*T* = 120 (2) K
0.3 × 0.3 × 0.25 mm

#### Data collection

Oxford Diffraction Xcalibur2+ CCD diffractometer
Absorption correction: multi-scan (*CrysAlis RED*; Oxford Diffraction, 2006 ▶) *T*_min_ = 0.823, *T*_max_ = 0.840
41227 measured reflections
23598 independent reflections
21012 reflections with *I* > 2σ(*I*)
*R*_int_ = 0.016

#### Refinement

*R*[*F* ^2^ > 2σ(*F* ^2^)] = 0.034
*wR*(*F* ^2^) = 0.086
*S* = 0.99
23591 reflections
2053 parameters
62 restraints
H atoms treated by a mixture of independent and constrained refinement
Δρ_max_ = 0.81 e Å^−3^
Δρ_min_ = −0.62 e Å^−3^
Absolute structure: Flack (1983 ▶), with 10782 Friedel pairs
Flack parameter: 0.105 (6)

### 

Data collection: *CrysAlis CCD* (Oxford Diffraction, 2006 ▶); cell refinement: *CrysAlis RED* (Oxford Diffraction, 2006 ▶); data reduction: *CrysAlis RED*; program(s) used to solve structure: *SHELXS97* (Sheldrick, 2008 ▶); program(s) used to refine structure: *SHELXL97* (Sheldrick, 2008 ▶); molecular graphics: *DIAMOND* (Brandenburg, 2006 ▶); software used to prepare material for publication: *SHELXL97*.

## Supplementary Material

Crystal structure: contains datablocks I, global. DOI: 10.1107/S1600536808001852/pk2079sup1.cif

Structure factors: contains datablocks I. DOI: 10.1107/S1600536808001852/pk2079Isup2.hkl

Additional supplementary materials:  crystallographic information; 3D view; checkCIF report

## Figures and Tables

**Table 1:** Hydrogen-bond geometry (Å, °)

*D*—H⋯*A*	*D*—H	H⋯*A*	*D*⋯*A*	*D*—H⋯*A*
O5—H5*W*⋯O85*W*	0.87 (4)	1.72 (2)	2.565 (3)	165 (4)
O85*W*—H85*V*⋯O97*W*	0.847 (19)	1.87 (2)	2.698 (4)	164 (4)
O85*W*—H85*W*⋯O93*W*	0.85 (4)	1.86 (2)	2.707 (4)	175 (4)
O93*W*—H93*V*⋯O16	0.843 (19)	1.92 (2)	2.745 (3)	166 (5)
O93*W*—H93*W*⋯O87*W*	0.84 (4)	1.88 (2)	2.710 (4)	176 (5)
O97*W*—H97*V*⋯O16	0.86 (4)	1.88 (2)	2.735 (3)	171 (4)
O97*W*—H97*W*⋯O74*W*	0.879 (19)	1.87 (2)	2.733 (4)	167 (4)
O87*W*—H87*W*⋯O6	0.838 (19)	2.03 (2)	2.868 (4)	177 (5)
O87*W*—H87*V*⋯O13	0.85 (4)	1.89 (2)	2.734 (4)	174 (5)
O74*W*—H74*V*⋯O91*W*	0.83 (2)	1.96 (2)	2.776 (4)	170 (5)
O74*W*—H74*W*⋯O83*W*	0.845 (19)	1.95 (3)	2.696 (5)	146 (5)
O91*W*—H91*V*⋯O80*W*	0.863 (19)	1.89 (2)	2.750 (4)	176 (5)
O91*W*—H91*W*⋯O79*W*	0.87 (4)	2.03 (2)	2.882 (4)	169 (4)
O80*W*—H80*V*⋯O15	0.83 (4)	2.03 (3)	2.796 (3)	154 (5)
O80*W*—H80*W*⋯O81*W*^i^	0.885 (19)	1.92 (2)	2.788 (4)	167 (4)
O98*W*—H98*V*⋯O15	0.819 (19)	1.97 (2)	2.789 (3)	176 (4)
O98*W*—H98*W*⋯O81*W*^i^	0.823 (19)	2.00 (3)	2.753 (4)	152 (4)
O81*W*—H81*W*⋯O12	0.85 (4)	1.98 (2)	2.820 (4)	170 (5)
O81*W*—H81*V*⋯O92*W*	0.825 (19)	1.88 (2)	2.692 (4)	169 (5)
O92*W*—H92*W*⋯O23	0.862 (19)	1.91 (2)	2.757 (4)	169 (4)
O92*W*—H92*V*⋯O99*W*	0.829 (19)	2.01 (2)	2.801 (4)	159 (4)
O99*W*—H99*W*⋯O85*W*	0.847 (19)	1.93 (2)	2.752 (4)	164 (4)
O99*W*—H99*V*⋯O96*W*	0.851 (19)	2.06 (3)	2.845 (5)	153 (4)
O96*W*—H96*W*⋯O24	0.96 (5)	1.75 (4)	2.533 (6)	137 (3)
O22—H22*V*⋯O14^ii^	0.86 (2)	1.67 (2)	2.518 (3)	167 (5)

Symmetry codes: (i) 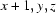; (ii) 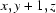.

**Fig. 1.**
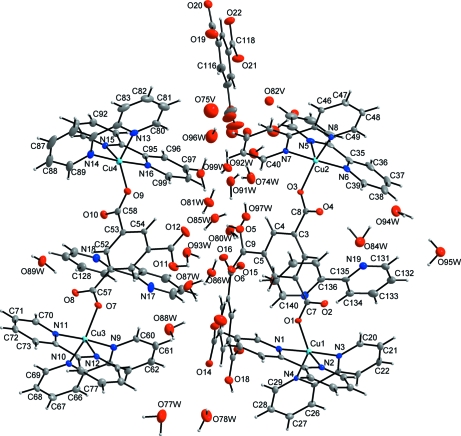
The molecular structure of the title complex. The non-H atoms are drawn as 40% probability displacement ellipsoids. Some of atom labels are omitted for clarity.

**Fig. 2.**
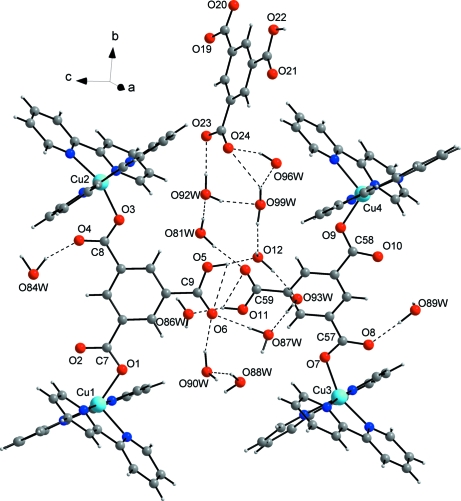
Part of the crystal structure showing the formation of O—H···O hydrogen bonds. Most of the water molecules of crystallization, and free Hbtc anions and bpy molecules have been omitted for clarity.

**Fig. 3.**
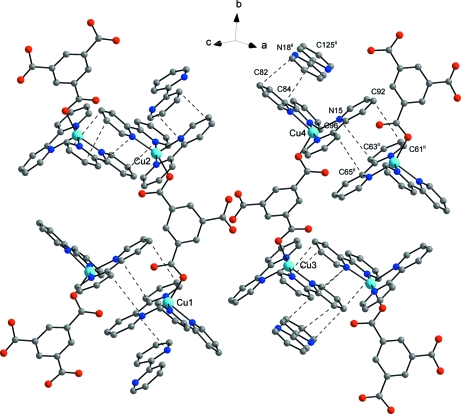
Part of the crystal structure showing the formation of π-π stacking interactions of the C···Cii and C···Nii type connecting free and coordinated bpy molecules [C84···C125ii = 3.544 (5) Å, C92···C61ii = 3.563 (5) Å, C96···C65ii = 3.425 (5) Å, C82···N18ii = 3.526 (6) Å and N15···C63ii = 3.510 (4) Å. Symmetry code: (ii) -x + 1, y + 1/2, -z]. The water molecules and Hbtc anions of crystallization have been omitted for clarity.

## supplementary crystallographic information

### Comment

The structure of the title complex (Fig. 1) comprises two crystallographically independent dinuclear Cu^II^ dications, two 1,3,5-carbotricarboxylate dianions (Hbtc), two 2,2'-bipyridine (bpy) and 26 water molecules of crystallization. The two Cu^II^ metal centres are bridged by one Hbtc anion in which two carboxylato groups are coordinated to two copper atoms in a monodentate fashion. Each of the metal centres is bonded to four N atoms of two bpy ligands and one O atom of the Hbtc ligand in a highly distorted square-pyramidal geometry (τ = 0.48 for Cu1, τ = 0.44 for Cu2, τ = 0.46 for Cu3 and τ = 0.46 for Cu4). The Cu—N distances vary from 1.966 (3) to 2.161 (3) Å, while those of Cu—O range from 1.987 (2) to 2.043 (2) Å. The atoms forming the Cu_2_(btc) moiety deviate from planarity with dihedral angles between the bridging benzene ring and the peripheral 4-membered C–O–Cu–O rings being in the range 18.02 (8)° to 28.20 (8)° (Brandenburg, 2006). The distance between the two Cu^II^ cations is 10.4977 (5) Å for Cu1···Cu2, and 10.5670 (5) Å for Cu3···Cu4. For comparison, the Cu···Cu separations in recently published Cu-complexes involving the Cu_2_(btc) moiety with one uncoordinated carboxylato group that are included in the CSD (Cambridge Structural Database, Version 5.28.1; Allen, 2002) are 9.5245 (5) Å (Pech & Pickardt, 1988), 9.6328 (10) Å (Chui *et al.*, 1999), 9.2316 (3) Å (Holmes *et al.*, 2004), 8.067 (7) and 8.207 (7) Å (Wang *et al.*, 2005), and 8.5541 (9) Å (Cooper *et al.*, 2005).

The secondary structure is stabilized by a variety of O—H···O hydrogen bonds (Table 1, Fig. 2) and π-π stacking interactions (Fig. 3), connecting the cations, Hbtc anions, and bpy and water molecules of crystallization.

### Experimental

The title complex was prepared in a quest to synthesize a trinuclear copper^II^ complex in which three metal centres would be connected *via* a benzene-1,3,5-tricarboxylato anion as a bridging ligand. Specifically: an aqueous solution (20 ml) of benzene-1,3,5-tricarboxylic acid (1 mmol) was added to a suspension of Cu(OH)_2_ (3 mmol) in water (40 ml). The reaction mixture was refluxed for 3 hrs. During this time period, an ethanolic solution of 2,2'-bipyridine (6 mmol) was added drop-wise to the reaction mixture while stirring. Then, the resulting solution was filtered and left to stand at room temperature. Blue crystals, suitable for single-crystal X-ray analysis, were obtained by slow evaporation of the solvents used over a period of several weeks.

### Refinement

Some atoms of water molecules of crystallization (namely O atoms O75, O82 and O83) were refined as disordered between two positions [the site occupancy factors were refined to 0.788 (4) and 0.212 (4)], where the major positions were refined anisotropically and the minor ones isotropically. C-bound H-atoms were included in the riding model approximation with C—H distances of 0.93 Å, and with *U*_iso_(H) values of 1.2*U*_eq_(C_aromatic_). The O-bound H atoms were refined, with the O—H distances restrained to 0.85 (2) Å and with *U*_iso_(H) values of 1.5*U*_eq_(O_water_); distances are given in Table 1. The maximum and minimum residual electron density peaks were located with distances 1.10 and 0.80 Å from the O24 atom.

A profile of a few (namely 7) low angle reflections has been strongly influenced by shielding by the beam-stopper, therefore these reflextions were excluded from the refinement.

0 - 1 3 5.03 10904.80 27.38 0.175 7.61 0 1 3 6.91 10664.59 27.30 0.173 7.61 1 2 1 55.69 2770.42 20.50 0.088 7.52 1 - 2 1 51.02 2605.64 20.14 0.085 7.52 - 1 -2 2 121.31 1069.63 13.08 0.055 7.09 - 1 -1 3 59.74 743.37 11.72 0.046 7.17 0 3 1 3202.81 5675.92 7.91 0.126 6.36

### Crystal data

**Table:**

[Cu_2_(C_9_H_4_O_6_)(C_10_H_8_N_2_)_4_](C_9_H_4_O_6_)·C_10_H_8_N_2_·13H_2_O	*F*_000_ = 3240
*M**_r_* = 1558.45	*D*_x_ = 1.465 Mg m^−^^3^
Monoclinic, *P*2_1_	Mo *K*α radiation λ = 0.71073 Å
Hall symbol: P 2yb	Cell parameters from 32974 reflections
*a* = 14.4638 (2) Å	θ = 2.6–32.0º
*b* = 19.7448 (2) Å	µ = 0.69 mm^−^^1^
*c* = 25.0638 (3) Å	*T* = 120 (2) K
β = 99.2051 (14)º	Prism, blue
*V* = 7065.66 (15) Å^3^	0.3 × 0.3 × 0.25 mm
*Z* = 4	

### Data collection

**Table:**

Oxford Diffraction Xcalibur2+ CCD diffractometer	23598 independent reflections
Radiation source: Enhance (Mo) X-ray Source	21012 reflections with *I* > 2σ(*I*)
Monochromator: graphite	*R*_int_ = 0.016
Detector resolution: 8.3611 pixels mm^-1^	θ_max_ = 25.1º
*T* = 293(2) K	θ_min_ = 2.7º
rotation method ω scans	*h* = −17→13
Absorption correction: multi-scan(CrysAlis RED; Oxford Diffraction, 2006)	*k* = −22→23
*T*_min_ = 0.823, *T*_max_ = 0.840	*l* = −29→29
41227 measured reflections	

### Refinement

**Table:**

Refinement on *F*^2^	Hydrogen site location: inferred from neighbouring sites
Least-squares matrix: full	H atoms treated by a mixture of independent and constrained refinement
*R*[*F*^2^ > 2σ(*F*^2^)] = 0.034	*w* = 1/[σ^2^(*F*_o_^2^) + (0.0475*P*)^2^ + 5*P*] where *P* = (*F*_o_^2^ + 2*F*_c_^2^)/3
*wR*(*F*^2^) = 0.086	(Δ/σ)_max_ < 0.001
*S* = 0.99	Δρ_max_ = 0.81 e Å^−^^3^
23591 reflections	Δρ_min_ = −0.62 e Å^−^^3^
2053 parameters	Extinction correction: none
62 restraints	Absolute structure: Flack (1983), 10782 Friedel pairs
Primary atom site location: structure-invariant direct methods	Flack parameter: 0.105 (6)
Secondary atom site location: difference Fourier map	

### Special details

**Table:**

Experimental. Absorption correction (full details): *CrysAlis RED* (Oxford Diffraction, 2006) Version 1.171.31.7 (release 18–10-2006 CrysAlis171. NET) (compiled Oct 18 2006,16:28:17). Empirical absorption correction using spherical harmonics, implemented in SCALE3 ABSPACK scaling algorithm.
Geometry. All e.s.d.'s (except the e.s.d. in the dihedral angle between two l.s. planes) are estimated using the full covariance matrix. The cell e.s.d.'s are taken into account individually in the estimation of e.s.d.'s in distances, angles and torsion angles; correlations between e.s.d.'s in cell parameters are only used when they are defined by crystal symmetry. An approximate (isotropic) treatment of cell e.s.d.'s is used for estimating e.s.d.'s involving l.s. planes.
Refinement. Refinement of *F*^2^ against ALL reflections. The weighted *R*-factor *wR* and goodness of fit *S* are based on *F*^2^, conventional *R*-factors *R* are based on *F*, with *F* set to zero for negative *F*^2^. The threshold expression of *F*^2^ > 2σ(*F*^2^) is used only for calculating *R*-factors(gt) *etc*. and is not relevant to the choice of reflections for refinement. *R*-factors based on *F*^2^ are statistically about twice as large as those based on *F*, and *R*- factors based on ALL data will be even larger.

### Fractional atomic coordinates and isotropic or equivalent isotropic displacement parameters (Å^2^)

**Table:**

	*x*	*y*	*z*	*U*_iso_*/*U*_eq_	Occ. (<1)
Cu1	0.31137 (2)	0.177710 (17)	0.498235 (14)	0.01341 (8)	
Cu2	0.18062 (2)	0.700682 (18)	0.498203 (14)	0.01502 (9)	
Cu3	0.17641 (2)	0.189610 (17)	−0.009072 (14)	0.01460 (9)	
Cu4	0.32895 (2)	0.712859 (19)	0.021961 (15)	0.01744 (9)	
O1	0.33861 (14)	0.26860 (11)	0.46507 (8)	0.0160 (5)	
O2	0.26838 (15)	0.30763 (11)	0.53194 (9)	0.0191 (5)	
O3	0.21388 (15)	0.61479 (11)	0.46425 (8)	0.0165 (5)	
O4	0.14569 (15)	0.55112 (11)	0.52073 (9)	0.0203 (5)	
O5	0.33234 (18)	0.52434 (12)	0.31428 (10)	0.0289 (6)	
H5W	0.357 (3)	0.535 (2)	0.2860 (12)	0.043*	
O6	0.34549 (17)	0.41255 (12)	0.30032 (9)	0.0268 (5)	
O7	0.15079 (15)	0.28225 (11)	0.01932 (9)	0.0189 (5)	
O8	0.22196 (15)	0.32671 (11)	−0.04501 (9)	0.0212 (5)	
O9	0.29559 (15)	0.62361 (11)	0.05025 (9)	0.0197 (5)	
O10	0.36466 (16)	0.56616 (12)	−0.00937 (9)	0.0234 (5)	
O11	0.1500 (2)	0.39334 (13)	0.18856 (11)	0.0408 (7)	
H11V	0.138 (3)	0.395 (2)	0.2209 (10)	0.061*	
O12	0.1995 (2)	0.49851 (14)	0.20408 (10)	0.0433 (7)	
O13	0.48939 (16)	0.27095 (12)	0.25271 (10)	0.0275 (5)	
O14	0.55947 (15)	0.17417 (11)	0.23701 (9)	0.0236 (5)	
O15	0.81789 (16)	0.48517 (12)	0.26154 (9)	0.0266 (5)	
O16	0.66177 (16)	0.48743 (11)	0.23960 (10)	0.0263 (5)	
O17	0.98334 (17)	0.26919 (13)	0.25050 (11)	0.0338 (6)	
O18	0.90396 (16)	0.17284 (12)	0.24242 (9)	0.0261 (5)	
H18V	0.957 (2)	0.152 (2)	0.2449 (19)	0.053 (14)*	
O19	−0.01066 (18)	1.02929 (14)	0.25877 (13)	0.0455 (7)	
O20	0.05922 (16)	1.12236 (12)	0.23636 (10)	0.0282 (5)	
O21	0.48534 (18)	1.02573 (13)	0.26211 (12)	0.0389 (6)	
O22	0.40450 (18)	1.12089 (13)	0.24599 (11)	0.0336 (6)	
H22V	0.459 (2)	1.134 (3)	0.240 (2)	0.069 (17)*	
O23	0.1576 (2)	0.81674 (15)	0.29295 (15)	0.0640 (10)	
O24	0.3095 (2)	0.81320 (16)	0.28853 (19)	0.0825 (13)	
H96W	0.363 (3)	0.812 (4)	0.2307 (14)	0.124*	
N1	0.19855 (17)	0.16962 (14)	0.44222 (10)	0.0162 (6)	
N2	0.22886 (18)	0.12282 (13)	0.54098 (10)	0.0153 (6)	
N3	0.42266 (17)	0.18089 (13)	0.55566 (9)	0.0145 (5)	
N4	0.40706 (17)	0.11554 (13)	0.46193 (10)	0.0134 (5)	
N5	0.27994 (19)	0.76810 (14)	0.47087 (11)	0.0211 (6)	
N6	0.28802 (18)	0.69145 (15)	0.55868 (10)	0.0214 (6)	
N7	0.07151 (17)	0.71179 (14)	0.44082 (9)	0.0148 (5)	
N8	0.09715 (17)	0.75529 (13)	0.54096 (10)	0.0136 (5)	
N9	0.29242 (17)	0.18288 (14)	0.04450 (10)	0.0177 (6)	
N10	0.25432 (18)	0.13091 (13)	−0.05250 (10)	0.0164 (6)	
N11	0.06172 (17)	0.18895 (14)	−0.06423 (10)	0.0161 (5)	
N12	0.08538 (18)	0.12943 (13)	0.03232 (10)	0.0150 (6)	
N13	0.24614 (19)	0.78209 (14)	0.06155 (12)	0.0224 (6)	
N14	0.21206 (18)	0.71632 (15)	−0.03157 (10)	0.0222 (6)	
N15	0.40951 (18)	0.76683 (14)	−0.02380 (10)	0.0164 (6)	
N16	0.44576 (17)	0.71712 (14)	0.07413 (10)	0.0166 (6)	
N17	0.7504 (2)	0.41281 (17)	0.06410 (11)	0.0314 (7)	
N18	0.7371 (2)	0.49218 (16)	−0.06644 (11)	0.0285 (7)	
N19	0.7651 (2)	0.48036 (14)	0.55385 (11)	0.0244 (6)	
N20	0.7330 (2)	0.39145 (15)	0.42675 (11)	0.0263 (7)	
C1	0.28239 (19)	0.38173 (15)	0.45829 (12)	0.0139 (6)	
C2	0.2410 (2)	0.43563 (15)	0.48184 (12)	0.0142 (6)	
H2	0.2232	0.4302	0.5156	0.017*	
C3	0.22635 (19)	0.49735 (15)	0.45522 (12)	0.0135 (6)	
C4	0.2508 (2)	0.50419 (15)	0.40398 (12)	0.0153 (6)	
H4	0.2398	0.5450	0.3856	0.018*	
C5	0.2916 (2)	0.45069 (15)	0.37996 (12)	0.0159 (6)	
C6	0.30695 (19)	0.38950 (15)	0.40747 (12)	0.0156 (6)	
H6	0.3339	0.3535	0.3916	0.019*	
C7	0.2982 (2)	0.31530 (16)	0.48793 (12)	0.0137 (6)	
C8	0.1907 (2)	0.55794 (17)	0.48279 (12)	0.0148 (7)	
C9	0.3247 (2)	0.46001 (16)	0.32702 (12)	0.0197 (7)	
C10	0.1911 (2)	0.19425 (16)	0.39166 (12)	0.0183 (7)	
H10	0.2419	0.2169	0.3815	0.022*	
C11	0.1105 (2)	0.18690 (17)	0.35450 (13)	0.0245 (7)	
H11	0.1074	0.2034	0.3195	0.029*	
C12	0.0343 (2)	0.15459 (18)	0.37027 (14)	0.0250 (8)	
H12	−0.0211	0.1498	0.3460	0.030*	
C13	0.0409 (2)	0.12944 (17)	0.42230 (13)	0.0220 (7)	
H13	−0.0098	0.1078	0.4336	0.026*	
C14	0.1252 (2)	0.13729 (15)	0.45743 (12)	0.0149 (7)	
C15	0.1420 (2)	0.11020 (16)	0.51299 (13)	0.0165 (7)	
C16	0.0764 (2)	0.07340 (17)	0.53608 (14)	0.0220 (7)	
H16	0.0171	0.0652	0.5167	0.026*	
C17	0.1004 (2)	0.04914 (18)	0.58816 (14)	0.0260 (8)	
H17	0.0571	0.0247	0.6041	0.031*	
C18	0.1883 (2)	0.06134 (17)	0.61621 (13)	0.0242 (7)	
H18	0.2053	0.0452	0.6513	0.029*	
C19	0.2521 (2)	0.09849 (16)	0.59123 (13)	0.0186 (7)	
H19	0.3120	0.1064	0.6100	0.022*	
C20	0.4272 (2)	0.21637 (16)	0.60201 (12)	0.0182 (7)	
H20	0.3739	0.2390	0.6088	0.022*	
C21	0.5071 (2)	0.22035 (17)	0.63955 (13)	0.0215 (7)	
H21	0.5078	0.2450	0.6712	0.026*	
C22	0.5866 (2)	0.18704 (18)	0.62942 (12)	0.0224 (7)	
H22	0.6419	0.1895	0.6541	0.027*	
C23	0.5831 (2)	0.15025 (17)	0.58247 (12)	0.0186 (7)	
H23	0.6362	0.1278	0.5750	0.022*	
C24	0.4995 (2)	0.14680 (16)	0.54614 (12)	0.0144 (6)	
C25	0.4881 (2)	0.10732 (15)	0.49526 (13)	0.0138 (7)	
C26	0.5565 (2)	0.06204 (17)	0.48350 (14)	0.0194 (7)	
H26	0.6127	0.0570	0.5070	0.023*	
C27	0.5387 (2)	0.02481 (17)	0.43599 (13)	0.0218 (7)	
H27	0.5829	−0.0058	0.4273	0.026*	
C28	0.4554 (2)	0.03353 (17)	0.40192 (13)	0.0216 (7)	
H28	0.4421	0.0090	0.3699	0.026*	
C29	0.3915 (2)	0.07969 (17)	0.41623 (13)	0.0189 (7)	
H29	0.3352	0.0860	0.3930	0.023*	
C30	0.2724 (3)	0.80565 (17)	0.42646 (16)	0.0289 (8)	
H30	0.2159	0.8054	0.4027	0.035*	
C31	0.3464 (3)	0.84595 (19)	0.41382 (18)	0.0395 (10)	
H31	0.3393	0.8721	0.3826	0.047*	
C32	0.4287 (3)	0.8452 (2)	0.44894 (19)	0.0413 (11)	
H32	0.4788	0.8711	0.4415	0.050*	
C33	0.4387 (3)	0.80710 (18)	0.49486 (18)	0.0351 (10)	
H33	0.4951	0.8067	0.5186	0.042*	
C34	0.3628 (2)	0.76860 (17)	0.50557 (15)	0.0245 (8)	
C35	0.3669 (2)	0.72547 (17)	0.55386 (14)	0.0234 (8)	
C36	0.4469 (2)	0.7193 (2)	0.59260 (15)	0.0310 (9)	
H36	0.5011	0.7432	0.5892	0.037*	
C37	0.4445 (3)	0.6771 (2)	0.63621 (15)	0.0374 (10)	
H37	0.4977	0.6717	0.6622	0.045*	
C38	0.3641 (3)	0.6430 (2)	0.64131 (15)	0.0363 (9)	
H38	0.3614	0.6152	0.6710	0.044*	
C39	0.2868 (2)	0.65089 (18)	0.60137 (13)	0.0250 (8)	
H39	0.2322	0.6272	0.6043	0.030*	
C40	0.0657 (2)	0.68812 (17)	0.39047 (12)	0.0200 (7)	
H40	0.1173	0.6662	0.3806	0.024*	
C41	−0.0141 (2)	0.69505 (18)	0.35280 (12)	0.0239 (7)	
H41	−0.0162	0.6781	0.3180	0.029*	
C42	−0.0912 (2)	0.72764 (17)	0.36742 (13)	0.0222 (7)	
H42	−0.1461	0.7324	0.3427	0.027*	
C43	−0.0856 (2)	0.75303 (16)	0.41935 (13)	0.0184 (7)	
H43	−0.1365	0.7750	0.4300	0.022*	
C44	−0.0027 (2)	0.74520 (16)	0.45529 (12)	0.0144 (6)	
C45	0.0126 (2)	0.77124 (15)	0.51142 (12)	0.0135 (6)	
C46	−0.0515 (2)	0.81177 (16)	0.53207 (13)	0.0162 (7)	
H46	−0.1088	0.8223	0.5113	0.019*	
C47	−0.0283 (2)	0.83638 (17)	0.58449 (13)	0.0210 (7)	
H47	−0.0705	0.8631	0.5994	0.025*	
C48	0.0578 (2)	0.82089 (17)	0.61413 (13)	0.0196 (7)	
H48	0.0752	0.8377	0.6490	0.023*	
C49	0.1176 (2)	0.77982 (16)	0.59080 (12)	0.0181 (7)	
H49	0.1752	0.7687	0.6111	0.022*	
C51	0.2132 (2)	0.39246 (15)	0.03421 (12)	0.0155 (6)	
C52	0.2586 (2)	0.44886 (15)	0.01607 (12)	0.0155 (6)	
H52	0.2758	0.4484	−0.0181	0.019*	
C53	0.27799 (19)	0.50543 (15)	0.04904 (12)	0.0146 (6)	
C54	0.2547 (2)	0.50496 (15)	0.10099 (12)	0.0157 (6)	
H54	0.2684	0.5424	0.1234	0.019*	
C55	0.2111 (2)	0.44889 (16)	0.11945 (12)	0.0166 (6)	
C56	0.1904 (2)	0.39272 (16)	0.08594 (12)	0.0169 (6)	
H56	0.1612	0.3552	0.0983	0.020*	
C57	0.1935 (2)	0.33025 (17)	−0.00082 (13)	0.0161 (7)	
C58	0.3175 (2)	0.56947 (17)	0.02816 (13)	0.0177 (7)	
C59	0.1858 (2)	0.44967 (16)	0.17488 (13)	0.0203 (7)	
C60	0.3033 (2)	0.20850 (17)	0.09471 (12)	0.0192 (7)	
H60	0.2542	0.2325	0.1056	0.023*	
C61	0.3850 (2)	0.20020 (18)	0.13051 (13)	0.0249 (7)	
H61	0.3907	0.2178	0.1653	0.030*	
C62	0.4587 (2)	0.16523 (18)	0.11405 (14)	0.0256 (8)	
H62	0.5150	0.1600	0.1374	0.031*	
C63	0.4475 (2)	0.13816 (18)	0.06249 (14)	0.0233 (8)	
H63	0.4960	0.1142	0.0509	0.028*	
C64	0.3629 (2)	0.14723 (16)	0.02837 (13)	0.0159 (7)	
C65	0.3415 (2)	0.11790 (16)	−0.02593 (13)	0.0166 (7)	
C66	0.4039 (2)	0.07867 (17)	−0.04936 (13)	0.0217 (7)	
H66	0.4638	0.0702	−0.0307	0.026*	
C67	0.3768 (3)	0.05244 (18)	−0.10018 (14)	0.0278 (8)	
H67	0.4179	0.0260	−0.1162	0.033*	
C68	0.2875 (2)	0.06586 (18)	−0.12723 (13)	0.0264 (8)	
H68	0.2678	0.0489	−0.1618	0.032*	
C69	0.2284 (2)	0.10487 (16)	−0.10186 (13)	0.0195 (7)	
H69	0.1681	0.1134	−0.1198	0.023*	
C70	0.0536 (2)	0.22199 (17)	−0.11147 (13)	0.0200 (7)	
H70	0.1035	0.2485	−0.1184	0.024*	
C71	−0.0253 (2)	0.21824 (18)	−0.15004 (13)	0.0230 (7)	
H71	−0.0283	0.2408	−0.1829	0.028*	
C72	−0.1001 (2)	0.18010 (18)	−0.13877 (12)	0.0219 (7)	
H72	−0.1546	0.1769	−0.1640	0.026*	
C73	−0.0933 (2)	0.14699 (17)	−0.08997 (13)	0.0205 (7)	
H73	−0.1435	0.1219	−0.0817	0.025*	
C74	−0.0111 (2)	0.15145 (16)	−0.05340 (12)	0.0154 (7)	
C75	0.0042 (2)	0.11586 (17)	−0.00025 (13)	0.0155 (7)	
C76	−0.0607 (2)	0.07034 (18)	0.01542 (14)	0.0219 (8)	
H76	−0.1169	0.0615	−0.0072	0.026*	
C77	−0.0399 (2)	0.03881 (19)	0.06496 (14)	0.0266 (8)	
H77	−0.0816	0.0077	0.0757	0.032*	
C78	0.0428 (2)	0.05361 (18)	0.09859 (13)	0.0237 (7)	
H78	0.0573	0.0333	0.1324	0.028*	
C79	0.1035 (2)	0.09946 (17)	0.08082 (12)	0.0189 (7)	
H79	0.1591	0.1099	0.1034	0.023*	
C80	0.2676 (3)	0.81166 (17)	0.10991 (14)	0.0268 (8)	
H80	0.3270	0.8047	0.1296	0.032*	
C81	0.2059 (3)	0.8519 (2)	0.13192 (18)	0.0407 (10)	
H81	0.2232	0.8717	0.1657	0.049*	
C82	0.1182 (3)	0.8621 (2)	0.10295 (18)	0.0431 (11)	
H82	0.0750	0.8888	0.1172	0.052*	
C83	0.0942 (3)	0.8329 (2)	0.05288 (18)	0.0384 (10)	
H83	0.0352	0.8397	0.0327	0.046*	
C84	0.1605 (2)	0.79250 (18)	0.03275 (15)	0.0262 (8)	
C85	0.1430 (2)	0.75727 (19)	−0.01989 (15)	0.0273 (9)	
C86	0.0596 (3)	0.7634 (2)	−0.05619 (16)	0.0386 (10)	
H86	0.0118	0.7914	−0.0482	0.046*	
C87	0.0489 (3)	0.7277 (3)	−0.10390 (18)	0.0518 (14)	
H87	−0.0057	0.7323	−0.1289	0.062*	
C88	0.1194 (3)	0.6849 (2)	−0.11476 (15)	0.0450 (12)	
H88	0.1124	0.6596	−0.1464	0.054*	
C89	0.2004 (3)	0.6809 (2)	−0.07726 (14)	0.0338 (9)	
H89	0.2484	0.6525	−0.0842	0.041*	
C90	0.3852 (2)	0.79141 (17)	−0.07358 (13)	0.0197 (7)	
H90	0.3260	0.7812	−0.0922	0.024*	
C91	0.4435 (2)	0.83120 (18)	−0.09883 (13)	0.0239 (8)	
H91	0.4241	0.8470	−0.1338	0.029*	
C92	0.5317 (2)	0.84730 (18)	−0.07097 (13)	0.0223 (7)	
H92	0.5723	0.8746	−0.0866	0.027*	
C93	0.5575 (2)	0.82168 (17)	−0.01930 (13)	0.0174 (7)	
H93	0.6164	0.8313	0.0000	0.021*	
C94	0.4959 (2)	0.78182 (16)	0.00354 (13)	0.0170 (7)	
C95	0.5169 (2)	0.75253 (16)	0.05826 (12)	0.0154 (7)	
C96	0.6020 (2)	0.75975 (17)	0.09243 (13)	0.0184 (7)	
H96	0.6504	0.7843	0.0813	0.022*	
C97	0.6140 (2)	0.73017 (18)	0.14300 (13)	0.0239 (8)	
H97	0.6709	0.7342	0.1660	0.029*	
C98	0.5412 (2)	0.69455 (18)	0.15920 (12)	0.0245 (7)	
H98	0.5477	0.6750	0.1934	0.029*	
C99	0.4581 (2)	0.68863 (18)	0.12335 (12)	0.0220 (7)	
H99	0.4092	0.6640	0.1338	0.026*	
C101	0.6516 (2)	0.27466 (16)	0.24506 (12)	0.0181 (7)	
C102	0.6532 (2)	0.34534 (16)	0.24653 (12)	0.0176 (7)	
H102	0.5976	0.3690	0.2466	0.021*	
C103	0.7364 (2)	0.38087 (17)	0.24787 (11)	0.0184 (7)	
C104	0.8185 (2)	0.34460 (16)	0.24774 (12)	0.0194 (7)	
H104	0.8747	0.3678	0.2488	0.023*	
C105	0.8185 (2)	0.27361 (16)	0.24606 (12)	0.0180 (6)	
C106	0.7344 (2)	0.23852 (17)	0.24439 (11)	0.0189 (7)	
H106	0.7335	0.1915	0.2428	0.023*	
C107	0.5593 (2)	0.23822 (16)	0.24485 (12)	0.0200 (7)	
C108	0.7384 (2)	0.45697 (17)	0.24959 (12)	0.0213 (7)	
C109	0.9096 (2)	0.23831 (16)	0.24654 (12)	0.0206 (7)	
C111	0.1526 (2)	1.02452 (17)	0.25587 (13)	0.0241 (7)	
C112	0.2361 (2)	1.05803 (17)	0.25242 (12)	0.0217 (7)	
H112	0.2357	1.1043	0.2456	0.026*	
C113	0.3201 (2)	1.02259 (17)	0.25911 (13)	0.0250 (7)	
C114	0.3199 (3)	0.95301 (17)	0.26913 (14)	0.0289 (8)	
H114	0.3759	0.9290	0.2733	0.035*	
C115	0.2363 (3)	0.91900 (18)	0.27289 (16)	0.0356 (9)	
C116	0.1534 (3)	0.95499 (18)	0.26585 (15)	0.0329 (9)	
H116	0.0975	0.9326	0.2678	0.039*	
C117	0.0606 (2)	1.06102 (18)	0.24976 (13)	0.0263 (8)	
C118	0.4105 (2)	1.05721 (18)	0.25579 (13)	0.0246 (8)	
C119	0.2339 (3)	0.8434 (2)	0.2850 (2)	0.0547 (13)	
C121	0.8128 (3)	0.4034 (2)	0.10910 (15)	0.0397 (10)	
H121	0.7936	0.3795	0.1373	0.048*	
C122	0.9034 (3)	0.4269 (2)	0.11615 (15)	0.0369 (9)	
H122	0.9442	0.4192	0.1482	0.044*	
C123	0.9320 (3)	0.4624 (2)	0.07414 (16)	0.0323 (9)	
H123	0.9927	0.4792	0.0774	0.039*	
C124	0.8693 (2)	0.47241 (18)	0.02750 (15)	0.0272 (8)	
H124	0.8874	0.4957	−0.0014	0.033*	
C125	0.7792 (2)	0.44778 (17)	0.02378 (13)	0.0224 (7)	
C126	0.7083 (2)	0.45810 (17)	−0.02582 (13)	0.0213 (7)	
C127	0.6175 (3)	0.43349 (18)	−0.02908 (15)	0.0283 (8)	
H127	0.5988	0.4112	0.0001	0.034*	
C128	0.5554 (3)	0.4428 (2)	−0.07660 (16)	0.0353 (9)	
H128	0.4942	0.4268	−0.0798	0.042*	
C129	0.5851 (3)	0.4758 (2)	−0.11905 (16)	0.0369 (9)	
H129	0.5455	0.4817	−0.1518	0.044*	
C130	0.6759 (3)	0.5001 (2)	−0.11146 (14)	0.0342 (9)	
H130	0.6954	0.5237	−0.1398	0.041*	
C131	0.8305 (3)	0.48761 (17)	0.59765 (15)	0.0297 (8)	
H131	0.8148	0.5119	0.6267	0.036*	
C132	0.9202 (3)	0.46121 (18)	0.60242 (16)	0.0334 (9)	
H132	0.9630	0.4671	0.6339	0.040*	
C133	0.9440 (3)	0.42613 (19)	0.55923 (16)	0.0317 (9)	
H133	1.0039	0.4083	0.5608	0.038*	
C134	0.8777 (2)	0.41781 (18)	0.51340 (14)	0.0248 (7)	
H134	0.8925	0.3946	0.4836	0.030*	
C135	0.7886 (2)	0.44463 (16)	0.51249 (14)	0.0199 (7)	
C136	0.7143 (2)	0.43522 (16)	0.46460 (13)	0.0199 (7)	
C137	0.6293 (2)	0.46906 (18)	0.46000 (14)	0.0266 (8)	
H137	0.6175	0.4981	0.4873	0.032*	
C138	0.5628 (3)	0.45949 (19)	0.41503 (15)	0.0316 (9)	
H138	0.5062	0.4828	0.4110	0.038*	
C139	0.5816 (3)	0.4147 (2)	0.37602 (14)	0.0331 (9)	
H139	0.5377	0.4067	0.3453	0.040*	
C140	0.6666 (3)	0.3821 (2)	0.38345 (14)	0.0331 (9)	
H140	0.6788	0.3519	0.3570	0.040*	
O74W	0.6909 (2)	0.71543 (17)	0.28672 (11)	0.0425 (7)	
H74V	0.728 (3)	0.721 (3)	0.2648 (16)	0.064*	
H74W	0.643 (2)	0.741 (2)	0.281 (2)	0.064*	
O75W	0.7064 (3)	0.90647 (19)	0.22531 (14)	0.0431 (10)	0.788 (4)
O75V	0.6244 (13)	0.8963 (10)	0.1900 (8)	0.080 (6)*	0.212 (4)
O76W	0.8487 (3)	0.86935 (19)	0.31107 (15)	0.0671 (10)	
H76W	0.867 (4)	0.902 (2)	0.3375 (19)	0.101*	
H76V	0.793 (3)	0.880 (3)	0.288 (2)	0.101*	
O77W	0.6843 (3)	0.0412 (2)	0.11924 (14)	0.0834 (13)	
H77W	0.686 (3)	−0.0053 (10)	0.119 (3)	0.125*	
H77V	0.659 (4)	0.035 (3)	0.0849 (8)	0.125*	
O78W	0.6635 (2)	0.04126 (19)	0.22631 (14)	0.0594 (9)	
H78W	0.675 (4)	0.0062 (11)	0.2092 (14)	0.089*	
H78V	0.608 (2)	0.049 (2)	0.233 (2)	0.089*	
O79W	0.9755 (2)	0.82287 (15)	0.25278 (11)	0.0400 (7)	
H79W	1.0384 (17)	0.811 (2)	0.2663 (18)	0.060*	
H79V	0.939 (3)	0.835 (2)	0.2757 (16)	0.060*	
O80W	0.90325 (19)	0.59599 (14)	0.21980 (11)	0.0367 (7)	
H80V	0.867 (3)	0.573 (2)	0.2350 (18)	0.055*	
H80W	0.9638 (15)	0.589 (2)	0.2304 (18)	0.055*	
O81W	0.08620 (19)	0.56713 (14)	0.26830 (11)	0.0351 (6)	
H81W	0.124 (3)	0.545 (2)	0.2526 (17)	0.053*	
H81V	0.112 (3)	0.6042 (14)	0.2737 (19)	0.053*	
O82W	0.6504 (2)	0.89441 (15)	0.38290 (11)	0.0296 (8)	0.788 (4)
O82V	0.5513 (10)	0.9154 (7)	0.3335 (6)	0.049 (4)*	0.212 (4)
O83W	0.5955 (3)	0.83319 (17)	0.28187 (13)	0.0370 (9)	0.788 (4)
O83V	0.5183 (10)	0.8217 (7)	0.2550 (5)	0.045 (4)*	0.212 (4)
O84W	0.1864 (2)	0.47151 (16)	0.61512 (12)	0.0518 (8)	
H84W	0.172 (4)	0.5093 (19)	0.6353 (14)	0.078*	
H84V	0.169 (4)	0.506 (2)	0.5918 (19)	0.078*	
O85W	0.42569 (17)	0.56689 (13)	0.24212 (10)	0.0294 (6)	
H85W	0.447 (3)	0.5410 (19)	0.2198 (14)	0.044*	
H85V	0.4769 (19)	0.583 (2)	0.2578 (16)	0.044*	
O86W	0.10880 (18)	0.37182 (13)	0.28346 (10)	0.0291 (5)	
H86W	0.074 (3)	0.3391 (16)	0.2757 (17)	0.044*	
H86V	0.075 (2)	0.3997 (18)	0.2960 (16)	0.044*	
O87W	0.38519 (19)	0.37109 (15)	0.19668 (11)	0.0371 (6)	
H87V	0.416 (3)	0.3382 (18)	0.2124 (17)	0.056*	
H87W	0.372 (3)	0.383 (2)	0.2266 (12)	0.056*	
O88W	0.1715 (2)	0.21905 (17)	0.18647 (11)	0.0437 (7)	
H88W	0.209 (3)	0.241 (2)	0.2127 (15)	0.066*	
H88V	0.137 (3)	0.191 (2)	0.2014 (18)	0.066*	
O89W	0.2289 (2)	0.41587 (16)	−0.12879 (11)	0.0439 (7)	
H89V	0.243 (3)	0.389 (2)	−0.1008 (14)	0.066*	
H89W	0.1676 (16)	0.422 (3)	−0.1432 (18)	0.066*	
O90W	0.3005 (2)	0.26278 (16)	0.28825 (14)	0.0529 (8)	
H90W	0.3632 (15)	0.256 (3)	0.2881 (18)	0.079*	
H90V	0.302 (3)	0.3081 (9)	0.296 (2)	0.079*	
O91W	0.82975 (19)	0.72420 (14)	0.22237 (11)	0.0364 (6)	
H91V	0.855 (3)	0.6846 (14)	0.2226 (19)	0.055*	
H91W	0.879 (2)	0.749 (2)	0.2318 (19)	0.055*	
O92W	0.19026 (18)	0.67920 (14)	0.29282 (10)	0.0303 (6)	
H92W	0.173 (3)	0.7211 (12)	0.2901 (18)	0.046*	
H92V	0.234 (2)	0.673 (2)	0.2761 (15)	0.046*	
O93W	0.49044 (18)	0.47794 (14)	0.17466 (10)	0.0324 (6)	
H93V	0.5431 (19)	0.474 (2)	0.1945 (15)	0.049*	
H93W	0.460 (3)	0.4440 (16)	0.1819 (18)	0.049*	
O94W	0.1747 (2)	0.55864 (17)	0.69522 (11)	0.0474 (7)	
H94W	0.141 (2)	0.540 (2)	0.7173 (18)	0.071*	
H94V	0.207 (2)	0.547 (3)	0.7268 (13)	0.071*	
O95W	0.0492 (2)	0.42768 (15)	0.82155 (12)	0.0420 (7)	
H95W	0.043 (3)	0.3924 (17)	0.8016 (17)	0.063*	
H95V	0.054 (3)	0.459 (2)	0.7967 (16)	0.063*	
O96W	0.4212 (3)	0.7970 (2)	0.22134 (15)	0.0695 (10)	
O97W	0.60019 (18)	0.59495 (13)	0.29311 (10)	0.0304 (6)	
H97V	0.624 (3)	0.5638 (17)	0.2757 (16)	0.046*	
H97W	0.629 (3)	0.6323 (15)	0.2858 (17)	0.046*	
O98W	0.99812 (17)	0.46804 (13)	0.31891 (10)	0.0293 (6)	
H98W	1.022 (3)	0.5047 (14)	0.3134 (17)	0.044*	
H98V	0.9443 (16)	0.473 (2)	0.3033 (16)	0.044*	
O99W	0.31100 (18)	0.67711 (14)	0.21588 (10)	0.0314 (6)	
H99W	0.337 (3)	0.6392 (14)	0.2242 (18)	0.047*	
H99V	0.329 (3)	0.7176 (12)	0.2229 (18)	0.047*	

### Atomic displacement parameters (Å^2^)

**Table:**

	*U*^11^	*U*^22^	*U*^33^	*U*^12^	*U*^13^	*U*^23^
Cu1	0.01105 (18)	0.0151 (2)	0.01389 (18)	0.00045 (14)	0.00131 (14)	0.00101 (14)
Cu2	0.01315 (18)	0.0166 (2)	0.01539 (17)	0.00278 (15)	0.00244 (14)	−0.00345 (15)
Cu3	0.01326 (18)	0.0163 (2)	0.01438 (17)	−0.00076 (14)	0.00261 (14)	−0.00261 (14)
Cu4	0.01484 (19)	0.0204 (2)	0.01707 (19)	−0.00415 (15)	0.00242 (15)	0.00289 (16)
O1	0.0160 (11)	0.0128 (11)	0.0195 (11)	0.0012 (9)	0.0032 (9)	0.0018 (9)
O2	0.0173 (11)	0.0208 (12)	0.0207 (12)	0.0019 (9)	0.0076 (9)	0.0056 (9)
O3	0.0192 (11)	0.0140 (11)	0.0166 (11)	0.0046 (9)	0.0043 (9)	−0.0014 (9)
O4	0.0199 (11)	0.0224 (12)	0.0202 (12)	0.0024 (9)	0.0083 (10)	−0.0016 (9)
O5	0.0427 (15)	0.0241 (13)	0.0233 (13)	0.0011 (11)	0.0162 (11)	0.0039 (10)
O6	0.0357 (14)	0.0242 (13)	0.0233 (12)	0.0048 (10)	0.0132 (11)	−0.0039 (10)
O7	0.0169 (11)	0.0171 (12)	0.0232 (12)	−0.0008 (9)	0.0051 (9)	−0.0050 (9)
O8	0.0240 (12)	0.0230 (12)	0.0184 (12)	−0.0024 (10)	0.0083 (10)	−0.0054 (9)
O9	0.0204 (12)	0.0183 (12)	0.0212 (12)	−0.0032 (9)	0.0056 (9)	−0.0011 (9)
O10	0.0222 (12)	0.0271 (13)	0.0219 (12)	−0.0067 (10)	0.0065 (10)	−0.0022 (10)
O11	0.070 (2)	0.0287 (15)	0.0314 (15)	−0.0088 (13)	0.0308 (14)	−0.0031 (12)
O12	0.070 (2)	0.0368 (16)	0.0274 (14)	−0.0198 (14)	0.0202 (14)	−0.0128 (12)
O13	0.0227 (13)	0.0228 (13)	0.0366 (14)	−0.0035 (10)	0.0031 (11)	0.0047 (10)
O14	0.0255 (12)	0.0183 (12)	0.0249 (12)	−0.0049 (9)	−0.0021 (9)	0.0015 (9)
O15	0.0297 (13)	0.0210 (12)	0.0289 (13)	−0.0064 (10)	0.0038 (11)	0.0006 (10)
O16	0.0240 (13)	0.0195 (12)	0.0346 (14)	−0.0014 (10)	0.0026 (11)	0.0038 (10)
O17	0.0241 (14)	0.0295 (14)	0.0473 (16)	−0.0034 (11)	0.0041 (12)	−0.0045 (12)
O18	0.0249 (13)	0.0250 (14)	0.0299 (13)	0.0003 (10)	0.0091 (10)	−0.0018 (10)
O19	0.0269 (14)	0.0354 (16)	0.075 (2)	−0.0094 (12)	0.0116 (14)	−0.0016 (14)
O20	0.0288 (13)	0.0269 (14)	0.0297 (13)	−0.0024 (11)	0.0075 (11)	−0.0016 (10)
O21	0.0316 (15)	0.0267 (14)	0.0599 (18)	0.0024 (12)	0.0121 (13)	0.0047 (12)
O22	0.0250 (14)	0.0253 (14)	0.0494 (16)	−0.0025 (11)	0.0025 (12)	0.0108 (12)
O23	0.058 (2)	0.0338 (18)	0.090 (3)	−0.0193 (15)	−0.0196 (18)	0.0213 (17)
O24	0.050 (2)	0.0294 (18)	0.154 (4)	−0.0003 (15)	−0.027 (2)	0.016 (2)
N1	0.0140 (13)	0.0182 (14)	0.0161 (13)	0.0016 (11)	0.0018 (10)	−0.0027 (11)
N2	0.0133 (14)	0.0142 (13)	0.0192 (14)	−0.0006 (11)	0.0053 (11)	−0.0027 (11)
N3	0.0156 (13)	0.0131 (13)	0.0153 (12)	−0.0013 (11)	0.0037 (10)	0.0026 (11)
N4	0.0144 (13)	0.0141 (13)	0.0118 (12)	−0.0004 (10)	0.0022 (10)	0.0030 (10)
N5	0.0205 (15)	0.0147 (14)	0.0306 (16)	0.0019 (11)	0.0117 (12)	−0.0035 (12)
N6	0.0169 (13)	0.0237 (16)	0.0234 (14)	0.0085 (12)	0.0027 (11)	−0.0109 (12)
N7	0.0157 (13)	0.0153 (14)	0.0139 (12)	0.0002 (11)	0.0039 (10)	−0.0018 (11)
N8	0.0131 (13)	0.0144 (14)	0.0135 (13)	−0.0002 (10)	0.0030 (10)	0.0000 (10)
N9	0.0167 (13)	0.0171 (14)	0.0201 (13)	−0.0032 (11)	0.0053 (10)	−0.0016 (11)
N10	0.0170 (14)	0.0172 (15)	0.0155 (13)	−0.0027 (11)	0.0039 (11)	−0.0025 (11)
N11	0.0174 (13)	0.0153 (14)	0.0168 (12)	0.0008 (11)	0.0063 (10)	−0.0026 (11)
N12	0.0147 (13)	0.0166 (14)	0.0149 (13)	0.0007 (11)	0.0057 (11)	−0.0027 (10)
N13	0.0204 (15)	0.0174 (15)	0.0307 (16)	−0.0009 (12)	0.0076 (12)	0.0063 (12)
N14	0.0162 (14)	0.0281 (16)	0.0213 (14)	−0.0096 (12)	0.0003 (11)	0.0052 (13)
N15	0.0142 (13)	0.0191 (14)	0.0159 (13)	0.0010 (11)	0.0021 (11)	0.0018 (11)
N16	0.0160 (13)	0.0158 (14)	0.0187 (13)	−0.0011 (11)	0.0045 (10)	0.0003 (11)
N17	0.0364 (18)	0.0425 (19)	0.0158 (14)	−0.0088 (15)	0.0055 (13)	0.0038 (13)
N18	0.0327 (17)	0.0318 (17)	0.0218 (15)	−0.0002 (13)	0.0066 (13)	0.0015 (13)
N19	0.0261 (16)	0.0214 (15)	0.0258 (16)	−0.0026 (12)	0.0041 (13)	0.0006 (12)
N20	0.0318 (17)	0.0295 (17)	0.0195 (15)	0.0072 (13)	0.0097 (13)	0.0025 (12)
C1	0.0095 (14)	0.0159 (15)	0.0158 (15)	−0.0024 (11)	0.0005 (12)	−0.0002 (12)
C2	0.0092 (14)	0.0175 (16)	0.0163 (15)	−0.0044 (11)	0.0037 (12)	−0.0018 (12)
C3	0.0077 (14)	0.0153 (15)	0.0173 (15)	−0.0010 (11)	0.0010 (12)	−0.0045 (12)
C4	0.0146 (15)	0.0149 (16)	0.0160 (15)	−0.0006 (12)	0.0013 (12)	0.0018 (12)
C5	0.0136 (15)	0.0195 (16)	0.0134 (15)	−0.0026 (12)	−0.0009 (12)	−0.0007 (12)
C6	0.0105 (14)	0.0148 (15)	0.0219 (16)	0.0011 (11)	0.0038 (12)	−0.0058 (12)
C7	0.0098 (15)	0.0127 (16)	0.0183 (15)	−0.0025 (12)	0.0008 (12)	0.0004 (12)
C8	0.0108 (15)	0.0163 (18)	0.0164 (16)	0.0014 (12)	−0.0008 (12)	−0.0003 (13)
C9	0.0216 (16)	0.0210 (17)	0.0163 (16)	−0.0020 (13)	0.0029 (13)	−0.0001 (13)
C10	0.0223 (16)	0.0168 (17)	0.0160 (14)	0.0016 (13)	0.0035 (12)	0.0013 (13)
C11	0.0301 (18)	0.0247 (19)	0.0179 (15)	0.0030 (15)	0.0016 (13)	−0.0003 (14)
C12	0.0197 (17)	0.0290 (19)	0.0235 (18)	0.0024 (14)	−0.0057 (14)	−0.0079 (14)
C13	0.0178 (17)	0.0229 (19)	0.0251 (18)	−0.0012 (14)	0.0026 (14)	−0.0115 (14)
C14	0.0141 (16)	0.0115 (16)	0.0191 (16)	0.0020 (12)	0.0024 (13)	−0.0038 (12)
C15	0.0131 (16)	0.0140 (17)	0.0233 (17)	0.0032 (12)	0.0053 (13)	−0.0029 (13)
C16	0.0187 (17)	0.0214 (18)	0.0271 (18)	−0.0047 (14)	0.0074 (14)	−0.0052 (14)
C17	0.0295 (19)	0.026 (2)	0.0260 (18)	−0.0045 (15)	0.0141 (15)	−0.0034 (15)
C18	0.0326 (19)	0.0218 (18)	0.0189 (17)	−0.0022 (15)	0.0061 (15)	0.0023 (14)
C19	0.0157 (16)	0.0208 (18)	0.0192 (17)	0.0014 (13)	0.0024 (13)	0.0011 (13)
C20	0.0167 (16)	0.0189 (17)	0.0196 (16)	0.0003 (13)	0.0050 (12)	0.0001 (13)
C21	0.0220 (17)	0.0258 (19)	0.0166 (16)	−0.0052 (14)	0.0029 (13)	−0.0004 (13)
C22	0.0167 (15)	0.032 (2)	0.0163 (15)	−0.0044 (14)	−0.0050 (12)	0.0043 (14)
C23	0.0119 (16)	0.0265 (18)	0.0167 (16)	−0.0004 (13)	0.0001 (12)	0.0072 (13)
C24	0.0127 (15)	0.0155 (16)	0.0153 (15)	−0.0048 (12)	0.0032 (12)	0.0080 (12)
C25	0.0138 (16)	0.0095 (16)	0.0189 (16)	−0.0014 (12)	0.0055 (13)	0.0071 (12)
C26	0.0147 (16)	0.0194 (18)	0.0253 (18)	0.0035 (13)	0.0061 (13)	0.0029 (14)
C27	0.0218 (17)	0.0198 (18)	0.0266 (18)	0.0051 (14)	0.0129 (14)	0.0024 (14)
C28	0.0246 (18)	0.0212 (18)	0.0207 (17)	0.0008 (14)	0.0093 (14)	−0.0021 (13)
C29	0.0178 (16)	0.0228 (18)	0.0167 (16)	−0.0034 (13)	0.0041 (13)	0.0013 (13)
C30	0.0269 (19)	0.0186 (18)	0.045 (2)	0.0008 (14)	0.0164 (17)	0.0002 (16)
C31	0.046 (3)	0.022 (2)	0.058 (3)	0.0045 (18)	0.031 (2)	0.0001 (18)
C32	0.029 (2)	0.027 (2)	0.072 (3)	−0.0032 (17)	0.024 (2)	−0.009 (2)
C33	0.0216 (19)	0.0213 (19)	0.066 (3)	−0.0018 (15)	0.0167 (19)	−0.0153 (19)
C34	0.0162 (17)	0.0188 (18)	0.040 (2)	0.0037 (14)	0.0079 (15)	−0.0161 (15)
C35	0.0164 (17)	0.0249 (19)	0.0284 (18)	0.0053 (14)	0.0024 (14)	−0.0148 (14)
C36	0.0177 (17)	0.034 (2)	0.039 (2)	0.0061 (15)	−0.0006 (15)	−0.0231 (18)
C37	0.027 (2)	0.054 (3)	0.0269 (19)	0.0171 (19)	−0.0075 (15)	−0.0204 (19)
C38	0.041 (2)	0.044 (2)	0.0229 (19)	0.0173 (19)	0.0004 (17)	−0.0114 (17)
C39	0.0271 (19)	0.030 (2)	0.0169 (17)	0.0104 (15)	0.0008 (14)	−0.0057 (14)
C40	0.0222 (16)	0.0220 (18)	0.0169 (15)	0.0043 (14)	0.0068 (12)	−0.0026 (14)
C41	0.0307 (18)	0.0258 (19)	0.0156 (15)	0.0019 (15)	0.0052 (13)	−0.0035 (14)
C42	0.0233 (17)	0.0255 (19)	0.0165 (16)	0.0014 (14)	−0.0003 (13)	0.0027 (13)
C43	0.0140 (16)	0.0187 (17)	0.0230 (17)	0.0014 (13)	0.0041 (13)	−0.0021 (13)
C44	0.0142 (16)	0.0132 (16)	0.0160 (15)	−0.0042 (12)	0.0031 (12)	−0.0024 (12)
C45	0.0143 (16)	0.0116 (16)	0.0152 (15)	−0.0029 (12)	0.0041 (12)	0.0013 (12)
C46	0.0131 (16)	0.0164 (17)	0.0191 (16)	0.0020 (13)	0.0030 (13)	−0.0024 (13)
C47	0.0186 (17)	0.0244 (18)	0.0214 (17)	0.0070 (14)	0.0079 (14)	−0.0053 (14)
C48	0.0215 (17)	0.0222 (18)	0.0155 (16)	0.0002 (14)	0.0044 (13)	−0.0058 (13)
C49	0.0185 (16)	0.0192 (17)	0.0162 (16)	−0.0007 (13)	0.0011 (13)	−0.0004 (13)
C51	0.0099 (14)	0.0175 (16)	0.0193 (16)	0.0015 (12)	0.0030 (12)	−0.0045 (12)
C52	0.0124 (15)	0.0196 (16)	0.0143 (16)	0.0025 (12)	0.0016 (13)	0.0028 (12)
C53	0.0074 (14)	0.0167 (16)	0.0191 (15)	0.0027 (11)	0.0002 (12)	0.0024 (12)
C54	0.0145 (15)	0.0143 (15)	0.0175 (15)	−0.0006 (12)	−0.0002 (12)	−0.0012 (12)
C55	0.0138 (15)	0.0215 (17)	0.0134 (15)	0.0028 (12)	−0.0017 (12)	−0.0010 (12)
C56	0.0116 (15)	0.0192 (16)	0.0202 (16)	0.0008 (12)	0.0033 (12)	0.0022 (12)
C57	0.0091 (15)	0.0176 (18)	0.0213 (17)	0.0032 (12)	0.0014 (13)	−0.0041 (13)
C58	0.0124 (15)	0.0212 (18)	0.0182 (17)	−0.0021 (13)	−0.0014 (13)	0.0032 (14)
C59	0.0201 (16)	0.0212 (17)	0.0188 (16)	−0.0006 (13)	0.0007 (13)	−0.0003 (13)
C60	0.0222 (16)	0.0196 (17)	0.0165 (15)	−0.0032 (14)	0.0055 (12)	−0.0043 (14)
C61	0.0258 (17)	0.0286 (19)	0.0184 (15)	−0.0050 (15)	−0.0018 (13)	−0.0011 (15)
C62	0.0219 (18)	0.028 (2)	0.0247 (18)	−0.0006 (14)	−0.0047 (14)	0.0006 (14)
C63	0.0164 (17)	0.027 (2)	0.0252 (18)	0.0000 (14)	−0.0006 (14)	0.0038 (15)
C64	0.0162 (16)	0.0128 (16)	0.0204 (16)	−0.0031 (13)	0.0079 (13)	0.0033 (13)
C65	0.0139 (16)	0.0144 (16)	0.0221 (17)	−0.0020 (13)	0.0050 (13)	0.0042 (13)
C66	0.0170 (17)	0.0247 (19)	0.0245 (18)	0.0049 (14)	0.0071 (14)	0.0044 (14)
C67	0.034 (2)	0.025 (2)	0.0279 (19)	0.0089 (16)	0.0157 (16)	−0.0045 (15)
C68	0.035 (2)	0.0275 (19)	0.0164 (17)	0.0044 (16)	0.0043 (15)	−0.0080 (14)
C69	0.0194 (17)	0.0214 (18)	0.0174 (16)	0.0010 (13)	0.0022 (13)	0.0003 (13)
C70	0.0222 (17)	0.0196 (18)	0.0200 (16)	0.0003 (13)	0.0087 (13)	0.0010 (13)
C71	0.0245 (17)	0.0279 (19)	0.0165 (16)	0.0054 (14)	0.0034 (13)	0.0022 (14)
C72	0.0176 (15)	0.0278 (19)	0.0187 (15)	0.0062 (14)	−0.0017 (12)	−0.0062 (14)
C73	0.0189 (17)	0.0234 (18)	0.0196 (17)	0.0020 (14)	0.0042 (13)	−0.0058 (14)
C74	0.0131 (15)	0.0153 (16)	0.0182 (16)	0.0015 (12)	0.0037 (12)	−0.0067 (12)
C75	0.0151 (16)	0.0182 (17)	0.0142 (15)	−0.0009 (13)	0.0048 (12)	−0.0057 (13)
C76	0.0160 (17)	0.0258 (19)	0.0246 (18)	−0.0046 (14)	0.0053 (14)	−0.0013 (15)
C77	0.0240 (19)	0.030 (2)	0.0278 (19)	−0.0049 (15)	0.0112 (15)	0.0023 (15)
C78	0.0273 (18)	0.0271 (19)	0.0178 (17)	0.0004 (15)	0.0067 (14)	−0.0005 (14)
C79	0.0188 (17)	0.0255 (18)	0.0123 (15)	0.0033 (13)	0.0026 (13)	−0.0028 (13)
C80	0.038 (2)	0.0184 (18)	0.0267 (19)	−0.0058 (15)	0.0115 (16)	−0.0012 (14)
C81	0.056 (3)	0.025 (2)	0.048 (2)	−0.0038 (19)	0.031 (2)	−0.0028 (18)
C82	0.056 (3)	0.025 (2)	0.057 (3)	0.0088 (19)	0.036 (2)	0.0039 (19)
C83	0.026 (2)	0.035 (2)	0.059 (3)	0.0102 (17)	0.0206 (19)	0.023 (2)
C84	0.0232 (18)	0.0238 (19)	0.034 (2)	−0.0031 (14)	0.0109 (15)	0.0145 (15)
C85	0.0151 (17)	0.034 (2)	0.032 (2)	−0.0070 (15)	0.0038 (15)	0.0205 (17)
C86	0.023 (2)	0.055 (3)	0.037 (2)	−0.0095 (18)	0.0024 (17)	0.026 (2)
C87	0.029 (2)	0.074 (3)	0.046 (3)	−0.025 (2)	−0.0123 (19)	0.038 (2)
C88	0.050 (3)	0.060 (3)	0.0224 (18)	−0.030 (2)	−0.0036 (17)	0.0150 (19)
C89	0.037 (2)	0.040 (2)	0.0225 (18)	−0.0148 (18)	−0.0006 (15)	0.0078 (17)
C90	0.0197 (17)	0.0225 (18)	0.0167 (16)	0.0016 (14)	0.0019 (13)	−0.0003 (13)
C91	0.0231 (18)	0.033 (2)	0.0157 (16)	0.0005 (15)	0.0033 (14)	0.0064 (14)
C92	0.0221 (18)	0.0255 (19)	0.0215 (17)	−0.0059 (14)	0.0103 (14)	0.0051 (14)
C93	0.0124 (16)	0.0223 (18)	0.0177 (16)	−0.0019 (13)	0.0034 (13)	−0.0004 (13)
C94	0.0176 (16)	0.0174 (17)	0.0158 (16)	0.0025 (13)	0.0015 (13)	−0.0004 (13)
C95	0.0163 (16)	0.0161 (16)	0.0148 (15)	0.0000 (13)	0.0058 (13)	−0.0002 (12)
C96	0.0165 (16)	0.0191 (17)	0.0196 (16)	−0.0004 (13)	0.0024 (13)	0.0019 (13)
C97	0.0196 (17)	0.029 (2)	0.0206 (17)	−0.0017 (14)	−0.0027 (13)	−0.0017 (14)
C98	0.0279 (17)	0.029 (2)	0.0151 (15)	−0.0020 (15)	−0.0006 (13)	0.0070 (14)
C99	0.0218 (16)	0.0262 (19)	0.0196 (15)	−0.0019 (14)	0.0078 (13)	0.0062 (14)
C101	0.0213 (17)	0.0219 (17)	0.0106 (14)	−0.0050 (13)	0.0007 (12)	0.0023 (12)
C102	0.0212 (17)	0.0197 (17)	0.0107 (15)	−0.0027 (13)	−0.0008 (12)	0.0010 (12)
C103	0.0280 (18)	0.0206 (17)	0.0058 (14)	−0.0028 (13)	0.0003 (12)	0.0017 (12)
C104	0.0252 (18)	0.0214 (17)	0.0118 (15)	−0.0073 (14)	0.0037 (13)	−0.0006 (12)
C105	0.0209 (16)	0.0239 (17)	0.0098 (14)	−0.0013 (13)	0.0037 (12)	0.0016 (12)
C106	0.0286 (18)	0.0182 (16)	0.0092 (14)	−0.0052 (13)	0.0006 (12)	0.0008 (11)
C107	0.0213 (17)	0.0221 (18)	0.0143 (16)	−0.0055 (14)	−0.0041 (13)	0.0027 (13)
C108	0.0279 (19)	0.0237 (18)	0.0133 (16)	−0.0089 (15)	0.0066 (14)	0.0017 (13)
C109	0.0278 (18)	0.0219 (18)	0.0121 (15)	−0.0038 (14)	0.0029 (13)	0.0001 (12)
C111	0.0300 (19)	0.0223 (18)	0.0192 (17)	−0.0068 (14)	0.0013 (14)	−0.0021 (13)
C112	0.0322 (19)	0.0173 (17)	0.0139 (16)	0.0008 (14)	−0.0012 (14)	0.0001 (12)
C113	0.0313 (19)	0.0224 (18)	0.0193 (17)	−0.0021 (15)	−0.0022 (14)	−0.0013 (13)
C114	0.0294 (19)	0.0185 (18)	0.034 (2)	0.0030 (15)	−0.0095 (16)	−0.0018 (15)
C115	0.043 (2)	0.0178 (18)	0.039 (2)	−0.0045 (16)	−0.0150 (18)	0.0059 (16)
C116	0.031 (2)	0.028 (2)	0.037 (2)	−0.0125 (16)	−0.0037 (17)	0.0036 (16)
C117	0.035 (2)	0.0235 (19)	0.0208 (17)	−0.0075 (16)	0.0055 (15)	−0.0044 (14)
C118	0.033 (2)	0.0253 (19)	0.0143 (16)	0.0077 (16)	0.0003 (14)	−0.0040 (14)
C119	0.043 (3)	0.033 (2)	0.079 (3)	0.000 (2)	−0.018 (2)	0.021 (2)
C121	0.044 (2)	0.054 (3)	0.0213 (19)	−0.010 (2)	0.0064 (17)	0.0076 (17)
C122	0.044 (2)	0.040 (2)	0.0235 (19)	0.0019 (19)	−0.0038 (17)	0.0035 (17)
C123	0.027 (2)	0.033 (2)	0.036 (2)	0.0006 (16)	0.0014 (16)	0.0020 (17)
C124	0.0277 (19)	0.026 (2)	0.0294 (19)	0.0030 (15)	0.0090 (16)	0.0014 (15)
C125	0.0290 (19)	0.0209 (18)	0.0182 (17)	0.0012 (14)	0.0065 (14)	−0.0031 (13)
C126	0.0308 (19)	0.0167 (17)	0.0177 (17)	0.0031 (14)	0.0075 (15)	−0.0051 (13)
C127	0.032 (2)	0.0231 (19)	0.031 (2)	−0.0033 (15)	0.0067 (16)	0.0053 (15)
C128	0.029 (2)	0.034 (2)	0.040 (2)	−0.0038 (16)	−0.0053 (17)	0.0027 (17)
C129	0.045 (2)	0.032 (2)	0.029 (2)	0.0045 (18)	−0.0056 (17)	−0.0016 (16)
C130	0.042 (2)	0.040 (2)	0.0217 (19)	0.0005 (18)	0.0077 (17)	0.0059 (16)
C131	0.038 (2)	0.0200 (18)	0.0296 (19)	0.0021 (15)	0.0016 (16)	−0.0039 (14)
C132	0.035 (2)	0.023 (2)	0.038 (2)	0.0021 (16)	−0.0086 (17)	−0.0001 (16)
C133	0.0249 (19)	0.024 (2)	0.045 (2)	−0.0002 (15)	0.0019 (17)	−0.0014 (17)
C134	0.0237 (18)	0.0209 (18)	0.0297 (19)	0.0032 (14)	0.0035 (15)	−0.0004 (14)
C135	0.0253 (17)	0.0103 (16)	0.0257 (18)	−0.0025 (13)	0.0087 (15)	0.0041 (13)
C136	0.0207 (17)	0.0181 (17)	0.0228 (17)	−0.0013 (13)	0.0094 (14)	0.0059 (13)
C137	0.0280 (19)	0.0222 (19)	0.0295 (19)	0.0023 (15)	0.0040 (16)	−0.0077 (15)
C138	0.0268 (19)	0.031 (2)	0.036 (2)	0.0073 (16)	0.0027 (16)	−0.0016 (16)
C139	0.036 (2)	0.040 (2)	0.0224 (18)	0.0062 (17)	−0.0002 (16)	0.0022 (16)
C140	0.043 (2)	0.038 (2)	0.0191 (18)	0.0087 (18)	0.0073 (16)	−0.0032 (15)
O74W	0.0464 (18)	0.052 (2)	0.0296 (14)	−0.0208 (15)	0.0070 (13)	−0.0018 (14)
O75W	0.052 (2)	0.052 (2)	0.0281 (19)	−0.0023 (18)	0.0150 (17)	0.0097 (16)
O76W	0.086 (3)	0.057 (2)	0.060 (2)	0.015 (2)	0.016 (2)	−0.0009 (18)
O77W	0.131 (4)	0.081 (3)	0.0348 (19)	0.018 (3)	0.003 (2)	0.0087 (19)
O78W	0.0385 (18)	0.082 (3)	0.055 (2)	0.0281 (17)	−0.0011 (15)	−0.0207 (18)
O79W	0.0447 (18)	0.0396 (17)	0.0369 (16)	−0.0110 (14)	0.0100 (13)	−0.0030 (13)
O80W	0.0329 (15)	0.0383 (16)	0.0360 (16)	−0.0142 (13)	−0.0038 (13)	0.0110 (12)
O81W	0.0341 (16)	0.0323 (15)	0.0417 (16)	−0.0113 (12)	0.0148 (12)	−0.0080 (12)
O82W	0.047 (2)	0.0215 (16)	0.0208 (16)	0.0057 (14)	0.0082 (14)	−0.0008 (12)
O83W	0.046 (2)	0.0302 (19)	0.0344 (19)	−0.0058 (15)	0.0050 (16)	0.0009 (15)
O84W	0.076 (2)	0.0434 (19)	0.0380 (17)	0.0052 (17)	0.0149 (16)	0.0052 (14)
O85W	0.0243 (13)	0.0348 (15)	0.0298 (14)	−0.0044 (11)	0.0066 (11)	0.0041 (11)
O86W	0.0278 (14)	0.0285 (14)	0.0330 (14)	−0.0046 (10)	0.0112 (11)	−0.0018 (11)
O87W	0.0288 (14)	0.0471 (17)	0.0363 (15)	0.0039 (12)	0.0082 (12)	0.0198 (13)
O88W	0.0409 (17)	0.056 (2)	0.0364 (16)	−0.0109 (14)	0.0119 (13)	0.0012 (14)
O89W	0.0492 (17)	0.0523 (19)	0.0280 (15)	0.0020 (15)	0.0001 (13)	0.0090 (13)
O90W	0.0528 (19)	0.0460 (19)	0.066 (2)	−0.0088 (16)	0.0295 (17)	−0.0074 (16)
O91W	0.0362 (16)	0.0374 (16)	0.0331 (14)	−0.0107 (12)	−0.0024 (12)	−0.0003 (12)
O92W	0.0289 (14)	0.0291 (14)	0.0348 (14)	−0.0035 (12)	0.0107 (11)	−0.0086 (12)
O93W	0.0294 (14)	0.0406 (16)	0.0282 (14)	−0.0071 (12)	0.0072 (11)	0.0049 (12)
O94W	0.0515 (18)	0.058 (2)	0.0343 (16)	0.0157 (16)	0.0124 (14)	−0.0052 (14)
O95W	0.0461 (17)	0.0419 (18)	0.0387 (17)	0.0065 (14)	0.0084 (14)	−0.0027 (13)
O96W	0.068 (3)	0.075 (3)	0.063 (2)	0.002 (2)	0.0051 (19)	0.0061 (19)
O97W	0.0291 (14)	0.0279 (15)	0.0335 (15)	0.0012 (11)	0.0030 (11)	−0.0036 (11)
O98W	0.0262 (14)	0.0350 (15)	0.0275 (14)	−0.0017 (12)	0.0071 (11)	0.0004 (11)
O99W	0.0298 (14)	0.0338 (15)	0.0298 (13)	0.0040 (12)	0.0023 (11)	0.0028 (12)

### Geometric parameters (Å, °)

**Table:**

Cu1—N3	1.981 (2)	C55—C59	1.492 (4)
Cu1—N1	1.982 (2)	C56—H56	0.9300
Cu1—N2	2.039 (3)	C60—C61	1.374 (4)
Cu1—O1	2.043 (2)	C60—H60	0.9300
Cu1—N4	2.157 (3)	C61—C62	1.388 (5)
Cu2—N7	1.970 (2)	C61—H61	0.9300
Cu2—O3	1.991 (2)	C62—C63	1.384 (5)
Cu2—N6	1.998 (3)	C62—H62	0.9300
Cu2—N8	2.045 (2)	C63—C64	1.388 (5)
Cu2—N5	2.149 (3)	C63—H63	0.9300
Cu3—N9	1.979 (2)	C64—C65	1.466 (4)
Cu3—N11	1.982 (2)	C65—C66	1.390 (5)
Cu3—O7	2.018 (2)	C66—C67	1.373 (5)
Cu3—N10	2.047 (3)	C66—H66	0.9300
Cu3—N12	2.158 (3)	C67—C68	1.385 (5)
Cu4—N16	1.966 (3)	C67—H67	0.9300
Cu4—N14	1.984 (3)	C68—C69	1.379 (5)
Cu4—O9	1.987 (2)	C68—H68	0.9300
Cu4—N15	2.058 (3)	C69—H69	0.9300
Cu4—N13	2.161 (3)	C70—C71	1.375 (4)
O1—C7	1.276 (4)	C70—H70	0.9300
O2—C7	1.256 (4)	C71—C72	1.384 (5)
O3—C8	1.280 (4)	C71—H71	0.9300
O4—C8	1.243 (4)	C72—C73	1.376 (5)
O5—C9	1.319 (4)	C72—H72	0.9300
O5—H5W	0.87 (4)	C73—C74	1.383 (4)
O6—C9	1.217 (4)	C73—H73	0.9300
O7—C57	1.279 (4)	C74—C75	1.491 (4)
O8—C57	1.244 (4)	C75—C76	1.400 (5)
O9—C58	1.267 (4)	C76—C77	1.378 (5)
O10—C58	1.249 (4)	C76—H76	0.9300
O11—C59	1.296 (4)	C77—C78	1.380 (5)
O11—H11V	0.859 (19)	C77—H77	0.9300
O12—C59	1.208 (4)	C78—C79	1.384 (5)
O13—C107	1.241 (4)	C78—H78	0.9300
O14—C107	1.280 (4)	C79—H79	0.9300
O15—C108	1.269 (4)	C80—C81	1.374 (5)
O16—C108	1.251 (4)	C80—H80	0.9300
O17—C109	1.218 (4)	C81—C82	1.373 (6)
O18—C109	1.298 (4)	C81—H81	0.9300
O18—H18V	0.870 (19)	C82—C83	1.374 (6)
O19—C117	1.258 (4)	C82—H82	0.9300
O20—C117	1.256 (4)	C83—C84	1.401 (5)
O21—C118	1.236 (4)	C83—H83	0.9300
O22—C118	1.281 (4)	C84—C85	1.477 (5)
O22—H22V	0.86 (2)	C85—C86	1.396 (5)
O23—C119	1.268 (6)	C86—C87	1.376 (7)
O24—C119	1.235 (6)	C86—H86	0.9300
N1—C14	1.344 (4)	C87—C88	1.384 (7)
N1—C10	1.345 (4)	C87—H87	0.9300
N2—C19	1.340 (4)	C88—C89	1.382 (5)
N2—C15	1.361 (4)	C88—H88	0.9300
N3—C20	1.349 (4)	C89—H89	0.9300
N3—C24	1.353 (4)	C90—C91	1.378 (5)
N4—C29	1.335 (4)	C90—H90	0.9300
N4—C25	1.336 (4)	C91—C92	1.390 (5)
N5—C30	1.327 (5)	C91—H91	0.9300
N5—C34	1.363 (4)	C92—C93	1.385 (5)
N6—C39	1.339 (4)	C92—H92	0.9300
N6—C35	1.346 (4)	C93—C94	1.380 (5)
N7—C40	1.336 (4)	C93—H93	0.9300
N7—C44	1.357 (4)	C94—C95	1.475 (4)
N8—C49	1.328 (4)	C95—C96	1.390 (4)
N8—C45	1.361 (4)	C96—C97	1.381 (5)
N9—C60	1.342 (4)	C96—H96	0.9300
N9—C64	1.353 (4)	C97—C98	1.381 (5)
N10—C69	1.337 (4)	C97—H97	0.9300
N10—C65	1.353 (4)	C98—C99	1.386 (4)
N11—C70	1.340 (4)	C98—H98	0.9300
N11—C74	1.350 (4)	C99—H99	0.9300
N12—C79	1.340 (4)	C101—C102	1.396 (4)
N12—C75	1.346 (4)	C101—C106	1.397 (4)
N13—C80	1.336 (5)	C101—C107	1.516 (4)
N13—C84	1.346 (4)	C102—C103	1.389 (4)
N14—C89	1.329 (5)	C102—H102	0.9300
N14—C85	1.353 (5)	C103—C104	1.387 (5)
N15—C90	1.332 (4)	C103—C108	1.503 (5)
N15—C94	1.357 (4)	C104—C105	1.402 (5)
N16—C99	1.342 (4)	C104—H104	0.9300
N16—C95	1.355 (4)	C105—C106	1.394 (4)
N17—C121	1.340 (5)	C105—C109	1.490 (5)
N17—C125	1.344 (4)	C106—H106	0.9300
N18—C130	1.327 (5)	C111—C112	1.392 (5)
N18—C126	1.341 (4)	C111—C116	1.395 (5)
N19—C131	1.337 (5)	C111—C117	1.499 (5)
N19—C135	1.342 (4)	C112—C113	1.389 (5)
N20—C140	1.342 (5)	C112—H112	0.9300
N20—C136	1.342 (4)	C113—C114	1.397 (5)
C1—C6	1.385 (4)	C113—C118	1.490 (5)
C1—C2	1.397 (4)	C114—C115	1.399 (5)
C1—C7	1.507 (4)	C114—H114	0.9300
C2—C3	1.389 (4)	C115—C116	1.381 (5)
C2—H2	0.9300	C115—C119	1.525 (6)
C3—C4	1.392 (4)	C116—H116	0.9300
C3—C8	1.513 (4)	C121—C122	1.375 (5)
C4—C5	1.392 (4)	C121—H121	0.9300
C4—H4	0.9300	C122—C123	1.382 (5)
C5—C6	1.391 (4)	C122—H122	0.9300
C5—C9	1.492 (4)	C123—C124	1.375 (5)
C6—H6	0.9300	C123—H123	0.9300
C10—C11	1.379 (4)	C124—C125	1.380 (5)
C10—H10	0.9300	C124—H124	0.9300
C11—C12	1.384 (5)	C125—C126	1.493 (5)
C11—H11	0.9300	C126—C127	1.391 (5)
C12—C13	1.384 (5)	C127—C128	1.385 (5)
C12—H12	0.9300	C127—H127	0.9300
C13—C14	1.394 (4)	C128—C129	1.375 (6)
C13—H13	0.9300	C128—H128	0.9300
C14—C15	1.475 (4)	C129—C130	1.382 (6)
C15—C16	1.392 (5)	C129—H129	0.9300
C16—C17	1.382 (5)	C130—H130	0.9300
C16—H16	0.9300	C131—C132	1.385 (5)
C17—C18	1.373 (5)	C131—H131	0.9300
C17—H17	0.9300	C132—C133	1.375 (5)
C18—C19	1.402 (5)	C132—H132	0.9300
C18—H18	0.9300	C133—C134	1.383 (5)
C19—H19	0.9300	C133—H133	0.9300
C20—C21	1.371 (4)	C134—C135	1.390 (5)
C20—H20	0.9300	C134—H134	0.9300
C21—C22	1.384 (5)	C135—C136	1.489 (5)
C21—H21	0.9300	C136—C137	1.388 (5)
C22—C23	1.377 (5)	C137—C138	1.373 (5)
C22—H22	0.9300	C137—H137	0.9300
C23—C24	1.394 (4)	C138—C139	1.377 (5)
C23—H23	0.9300	C138—H138	0.9300
C24—C25	1.481 (4)	C139—C140	1.373 (5)
C25—C26	1.400 (4)	C139—H139	0.9300
C26—C27	1.388 (5)	C140—H140	0.9300
C26—H26	0.9300	O74W—H74V	0.83 (2)
C27—C28	1.372 (5)	O74W—H74W	0.845 (19)
C27—H27	0.9300	O75W—O75V	1.376 (19)
C28—C29	1.386 (5)	O76W—H76W	0.93 (5)
C28—H28	0.9300	O76W—H76V	0.93 (5)
C29—H29	0.9300	O77W—H77W	0.919 (19)
C30—C31	1.410 (5)	O77W—H77V	0.887 (19)
C30—H30	0.9300	O78W—H78W	0.846 (19)
C31—C32	1.363 (6)	O78W—H78V	0.860 (17)
C31—H31	0.9300	O79W—H79W	0.948 (19)
C32—C33	1.363 (6)	O79W—H79V	0.87 (4)
C32—H32	0.9300	O80W—H80V	0.83 (4)
C33—C34	1.397 (5)	O80W—H80W	0.885 (19)
C33—H33	0.9300	O81W—H81W	0.85 (4)
C34—C35	1.473 (5)	O81W—H81V	0.825 (19)
C35—C36	1.392 (5)	O82W—O82V	1.785 (14)
C36—C37	1.380 (6)	O83W—O83V	1.229 (14)
C36—H36	0.9300	O83V—O96W	1.596 (14)
C37—C38	1.367 (6)	O84W—H84W	0.94 (4)
C37—H37	0.9300	O84W—H84V	0.90 (2)
C38—C39	1.386 (5)	O85W—H85W	0.85 (4)
C38—H38	0.9300	O85W—H85V	0.847 (19)
C39—H39	0.9300	O86W—H86W	0.82 (4)
C40—C41	1.376 (4)	O86W—H86V	0.834 (19)
C40—H40	0.9300	O87W—H87V	0.85 (4)
C41—C42	1.388 (5)	O87W—H87W	0.838 (19)
C41—H41	0.9300	O88W—H88W	0.90 (2)
C42—C43	1.385 (5)	O88W—H88V	0.87 (4)
C42—H42	0.9300	O89W—H89V	0.88 (2)
C43—C44	1.389 (4)	O89W—H89W	0.909 (19)
C43—H43	0.9300	O90W—H90W	0.918 (18)
C44—C45	1.481 (4)	O90W—H90V	0.912 (16)
C45—C46	1.386 (4)	O91W—H91V	0.863 (19)
C46—C47	1.391 (4)	O91W—H91W	0.87 (4)
C46—H46	0.9300	O92W—H92W	0.862 (19)
C47—C48	1.378 (5)	O92W—H92V	0.829 (19)
C47—H47	0.9300	O93W—H93V	0.843 (19)
C48—C49	1.381 (4)	O93W—H93W	0.84 (4)
C48—H48	0.9300	O94W—H94W	0.88 (4)
C49—H49	0.9300	O94W—H94V	0.884 (18)
C51—C56	1.388 (4)	O95W—H95W	0.85 (4)
C51—C52	1.405 (4)	O95W—H95V	0.88 (2)
C51—C57	1.510 (4)	O96W—H96W	0.96 (5)
C52—C53	1.391 (4)	O97W—H97V	0.86 (4)
C52—H52	0.9300	O97W—H97W	0.879 (19)
C53—C54	1.396 (4)	O98W—H98W	0.823 (19)
C53—C58	1.515 (4)	O98W—H98V	0.819 (19)
C54—C55	1.389 (4)	O99W—H99W	0.847 (19)
C54—H54	0.9300	O99W—H99V	0.851 (19)
C55—C56	1.394 (4)		
			
N3—Cu1—N1	176.92 (12)	O8—C57—C51	120.3 (3)
N3—Cu1—N2	96.37 (10)	O7—C57—C51	115.6 (3)
N1—Cu1—N2	81.06 (11)	O10—C58—O9	125.3 (3)
N3—Cu1—O1	94.62 (9)	O10—C58—C53	119.9 (3)
N1—Cu1—O1	88.45 (10)	O9—C58—C53	114.7 (3)
N2—Cu1—O1	148.02 (9)	O12—C59—O11	124.0 (3)
N3—Cu1—N4	79.80 (10)	O12—C59—C55	122.1 (3)
N1—Cu1—N4	99.58 (10)	O11—C59—C55	113.9 (3)
N2—Cu1—N4	112.84 (10)	N9—C60—C61	121.8 (3)
O1—Cu1—N4	98.61 (9)	N9—C60—H60	119.1
N7—Cu2—O3	90.17 (10)	C61—C60—H60	119.1
N7—Cu2—N6	177.44 (10)	C60—C61—C62	119.1 (3)
O3—Cu2—N6	92.00 (10)	C60—C61—H61	120.4
N7—Cu2—N8	81.53 (10)	C62—C61—H61	120.4
O3—Cu2—N8	151.22 (10)	C63—C62—C61	119.3 (3)
N6—Cu2—N8	95.91 (10)	C63—C62—H62	120.3
N7—Cu2—N5	101.33 (11)	C61—C62—H62	120.3
O3—Cu2—N5	99.90 (10)	C62—C63—C64	119.0 (3)
N6—Cu2—N5	79.64 (11)	C62—C63—H63	120.5
N8—Cu2—N5	108.73 (10)	C64—C63—H63	120.5
N9—Cu3—N11	175.57 (12)	N9—C64—C63	121.0 (3)
N9—Cu3—O7	90.30 (10)	N9—C64—C65	115.2 (3)
N11—Cu3—O7	94.10 (10)	C63—C64—C65	123.7 (3)
N9—Cu3—N10	81.01 (10)	N10—C65—C66	121.0 (3)
N11—Cu3—N10	95.50 (10)	N10—C65—C64	115.1 (3)
O7—Cu3—N10	148.03 (10)	C66—C65—C64	123.9 (3)
N9—Cu3—N12	98.81 (10)	C67—C66—C65	119.6 (3)
N11—Cu3—N12	79.91 (10)	C67—C66—H66	120.2
O7—Cu3—N12	99.73 (9)	C65—C66—H66	120.2
N10—Cu3—N12	111.96 (10)	C66—C67—C68	119.2 (3)
N16—Cu4—N14	175.50 (13)	C66—C67—H67	120.4
N16—Cu4—O9	91.86 (10)	C68—C67—H67	120.4
N14—Cu4—O9	92.44 (10)	C69—C68—C67	118.7 (3)
N16—Cu4—N15	81.11 (10)	C69—C68—H68	120.7
N14—Cu4—N15	95.88 (10)	C67—C68—H68	120.7
O9—Cu4—N15	148.13 (10)	N10—C69—C68	122.6 (3)
N16—Cu4—N13	98.51 (11)	N10—C69—H69	118.7
N14—Cu4—N13	79.27 (11)	C68—C69—H69	118.7
O9—Cu4—N13	102.30 (10)	N11—C70—C71	122.7 (3)
N15—Cu4—N13	109.46 (11)	N11—C70—H70	118.7
C7—O1—Cu1	108.67 (18)	C71—C70—H70	118.7
C8—O3—Cu2	119.78 (19)	C70—C71—C72	118.3 (3)
C9—O5—H5W	119 (3)	C70—C71—H71	120.8
C57—O7—Cu3	113.90 (19)	C72—C71—H71	120.8
C58—O9—Cu4	120.0 (2)	C73—C72—C71	119.5 (3)
C59—O11—H11V	111 (3)	C73—C72—H72	120.2
C109—O18—H18V	115 (3)	C71—C72—H72	120.2
C118—O22—H22V	107 (4)	C72—C73—C74	119.3 (3)
C14—N1—C10	119.4 (3)	C72—C73—H73	120.3
C14—N1—Cu1	115.8 (2)	C74—C73—H73	120.3
C10—N1—Cu1	124.8 (2)	N11—C74—C73	121.2 (3)
C19—N2—C15	119.4 (3)	N11—C74—C75	115.4 (3)
C19—N2—Cu1	127.2 (2)	C73—C74—C75	123.4 (3)
C15—N2—Cu1	113.4 (2)	N12—C75—C76	121.1 (3)
C20—N3—C24	118.8 (3)	N12—C75—C74	116.0 (3)
C20—N3—Cu1	124.3 (2)	C76—C75—C74	122.9 (3)
C24—N3—Cu1	116.8 (2)	C77—C76—C75	118.9 (3)
C29—N4—C25	118.9 (3)	C77—C76—H76	120.6
C29—N4—Cu1	129.2 (2)	C75—C76—H76	120.6
C25—N4—Cu1	111.3 (2)	C76—C77—C78	119.8 (3)
C30—N5—C34	118.5 (3)	C76—C77—H77	120.1
C30—N5—Cu2	130.1 (2)	C78—C77—H77	120.1
C34—N5—Cu2	111.4 (2)	C77—C78—C79	118.4 (3)
C39—N6—C35	119.4 (3)	C77—C78—H78	120.8
C39—N6—Cu2	123.5 (2)	C79—C78—H78	120.8
C35—N6—Cu2	117.0 (2)	N12—C79—C78	122.6 (3)
C40—N7—C44	119.3 (3)	N12—C79—H79	118.7
C40—N7—Cu2	124.9 (2)	C78—C79—H79	118.7
C44—N7—Cu2	115.73 (19)	N13—C80—C81	123.0 (4)
C49—N8—C45	118.2 (3)	N13—C80—H80	118.5
C49—N8—Cu2	128.6 (2)	C81—C80—H80	118.5
C45—N8—Cu2	113.0 (2)	C82—C81—C80	118.5 (4)
C60—N9—C64	119.7 (3)	C82—C81—H81	120.7
C60—N9—Cu3	124.8 (2)	C80—C81—H81	120.7
C64—N9—Cu3	115.5 (2)	C81—C82—C83	119.8 (4)
C69—N10—C65	118.9 (3)	C81—C82—H82	120.1
C69—N10—Cu3	127.8 (2)	C83—C82—H82	120.1
C65—N10—Cu3	113.2 (2)	C82—C83—C84	118.7 (4)
C70—N11—C74	118.9 (3)	C82—C83—H83	120.6
C70—N11—Cu3	123.9 (2)	C84—C83—H83	120.6
C74—N11—Cu3	117.1 (2)	N13—C84—C83	121.2 (4)
C79—N12—C75	119.2 (3)	N13—C84—C85	114.7 (3)
C79—N12—Cu3	129.3 (2)	C83—C84—C85	124.1 (3)
C75—N12—Cu3	111.0 (2)	N14—C85—C86	120.2 (4)
C80—N13—C84	118.7 (3)	N14—C85—C84	116.6 (3)
C80—N13—Cu4	128.9 (2)	C86—C85—C84	123.2 (4)
C84—N13—Cu4	112.4 (2)	C87—C86—C85	119.1 (4)
C89—N14—C85	120.3 (3)	C87—C86—H86	120.4
C89—N14—Cu4	122.8 (3)	C85—C86—H86	120.4
C85—N14—Cu4	116.9 (2)	C86—C87—C88	120.0 (4)
C90—N15—C94	118.5 (3)	C86—C87—H87	120.0
C90—N15—Cu4	128.5 (2)	C88—C87—H87	120.0
C94—N15—Cu4	112.9 (2)	C89—C88—C87	118.2 (4)
C99—N16—C95	119.4 (3)	C89—C88—H88	120.9
C99—N16—Cu4	124.6 (2)	C87—C88—H88	120.9
C95—N16—Cu4	116.0 (2)	N14—C89—C88	122.2 (4)
C121—N17—C125	117.2 (3)	N14—C89—H89	118.9
C130—N18—C126	117.3 (3)	C88—C89—H89	118.9
C131—N19—C135	117.2 (3)	N15—C90—C91	123.3 (3)
C140—N20—C136	117.4 (3)	N15—C90—H90	118.4
C6—C1—C2	119.6 (3)	C91—C90—H90	118.4
C6—C1—C7	120.6 (3)	C90—C91—C92	118.6 (3)
C2—C1—C7	119.8 (3)	C90—C91—H91	120.7
C3—C2—C1	120.5 (3)	C92—C91—H91	120.7
C3—C2—H2	119.7	C93—C92—C91	118.4 (3)
C1—C2—H2	119.7	C93—C92—H92	120.8
C2—C3—C4	119.1 (3)	C91—C92—H92	120.8
C2—C3—C8	120.8 (3)	C94—C93—C92	120.0 (3)
C4—C3—C8	120.0 (3)	C94—C93—H93	120.0
C3—C4—C5	120.8 (3)	C92—C93—H93	120.0
C3—C4—H4	119.6	N15—C94—C93	121.2 (3)
C5—C4—H4	119.6	N15—C94—C95	114.8 (3)
C6—C5—C4	119.3 (3)	C93—C94—C95	124.0 (3)
C6—C5—C9	119.9 (3)	N16—C95—C96	120.8 (3)
C4—C5—C9	120.6 (3)	N16—C95—C94	115.0 (3)
C1—C6—C5	120.5 (3)	C96—C95—C94	124.3 (3)
C1—C6—H6	119.7	C97—C96—C95	119.4 (3)
C5—C6—H6	119.7	C97—C96—H96	120.3
O2—C7—O1	123.6 (3)	C95—C96—H96	120.3
O2—C7—C1	119.4 (3)	C98—C97—C96	119.7 (3)
O1—C7—C1	116.9 (3)	C98—C97—H97	120.1
O4—C8—O3	124.9 (3)	C96—C97—H97	120.1
O4—C8—C3	121.5 (3)	C97—C98—C99	118.4 (3)
O3—C8—C3	113.6 (3)	C97—C98—H98	120.8
O6—C9—O5	124.8 (3)	C99—C98—H98	120.8
O6—C9—C5	122.5 (3)	N16—C99—C98	122.4 (3)
O5—C9—C5	112.7 (3)	N16—C99—H99	118.8
N1—C10—C11	122.0 (3)	C98—C99—H99	118.8
N1—C10—H10	119.0	C102—C101—C106	120.0 (3)
C11—C10—H10	119.0	C102—C101—C107	119.0 (3)
C10—C11—C12	118.8 (3)	C106—C101—C107	121.0 (3)
C10—C11—H11	120.6	C103—C102—C101	121.1 (3)
C12—C11—H11	120.6	C103—C102—H102	119.5
C13—C12—C11	119.8 (3)	C101—C102—H102	119.5
C13—C12—H12	120.1	C104—C103—C102	118.6 (3)
C11—C12—H12	120.1	C104—C103—C108	120.2 (3)
C12—C13—C14	118.4 (3)	C102—C103—C108	121.2 (3)
C12—C13—H13	120.8	C103—C104—C105	121.4 (3)
C14—C13—H13	120.8	C103—C104—H104	119.3
N1—C14—C13	121.6 (3)	C105—C104—H104	119.3
N1—C14—C15	115.0 (3)	C106—C105—C104	119.6 (3)
C13—C14—C15	123.4 (3)	C106—C105—C109	122.3 (3)
N2—C15—C16	121.1 (3)	C104—C105—C109	118.1 (3)
N2—C15—C14	114.8 (3)	C105—C106—C101	119.4 (3)
C16—C15—C14	124.2 (3)	C105—C106—H106	120.3
C17—C16—C15	119.3 (3)	C101—C106—H106	120.3
C17—C16—H16	120.4	O13—C107—O14	124.1 (3)
C15—C16—H16	120.4	O13—C107—C101	119.4 (3)
C18—C17—C16	119.7 (3)	O14—C107—C101	116.5 (3)
C18—C17—H17	120.2	O16—C108—O15	125.2 (3)
C16—C17—H17	120.2	O16—C108—C103	117.5 (3)
C17—C18—C19	119.0 (3)	O15—C108—C103	117.3 (3)
C17—C18—H18	120.5	O17—C109—O18	123.3 (3)
C19—C18—H18	120.5	O17—C109—C105	121.9 (3)
N2—C19—C18	121.5 (3)	O18—C109—C105	114.9 (3)
N2—C19—H19	119.2	C112—C111—C116	119.7 (3)
C18—C19—H19	119.2	C112—C111—C117	122.0 (3)
N3—C20—C21	122.7 (3)	C116—C111—C117	118.3 (3)
N3—C20—H20	118.7	C113—C112—C111	120.3 (3)
C21—C20—H20	118.7	C113—C112—H112	119.9
C20—C21—C22	118.8 (3)	C111—C112—H112	119.9
C20—C21—H21	120.6	C112—C113—C114	119.3 (3)
C22—C21—H21	120.6	C112—C113—C118	121.4 (3)
C23—C22—C21	119.3 (3)	C114—C113—C118	119.3 (3)
C23—C22—H22	120.4	C113—C114—C115	120.8 (3)
C21—C22—H22	120.4	C113—C114—H114	119.6
C22—C23—C24	119.6 (3)	C115—C114—H114	119.6
C22—C23—H23	120.2	C116—C115—C114	119.1 (3)
C24—C23—H23	120.2	C116—C115—C119	118.9 (4)
N3—C24—C23	120.8 (3)	C114—C115—C119	122.0 (4)
N3—C24—C25	115.6 (3)	C115—C116—C111	120.8 (3)
C23—C24—C25	123.7 (3)	C115—C116—H116	119.6
N4—C25—C26	121.7 (3)	C111—C116—H116	119.6
N4—C25—C24	116.1 (3)	O20—C117—O19	123.3 (3)
C26—C25—C24	122.1 (3)	O20—C117—C111	117.9 (3)
C27—C26—C25	118.6 (3)	O19—C117—C111	118.8 (3)
C27—C26—H26	120.7	O21—C118—O22	123.3 (3)
C25—C26—H26	120.7	O21—C118—C113	121.4 (3)
C28—C27—C26	119.4 (3)	O22—C118—C113	115.3 (3)
C28—C27—H27	120.3	O24—C119—O23	124.8 (4)
C26—C27—H27	120.3	O24—C119—C115	116.0 (4)
C27—C28—C29	118.5 (3)	O23—C119—C115	119.1 (4)
C27—C28—H28	120.7	N17—C121—C122	124.1 (4)
C29—C28—H28	120.7	N17—C121—H121	117.9
N4—C29—C28	122.9 (3)	C122—C121—H121	117.9
N4—C29—H29	118.6	C121—C122—C123	117.9 (3)
C28—C29—H29	118.6	C121—C122—H122	121.0
N5—C30—C31	122.7 (4)	C123—C122—H122	121.0
N5—C30—H30	118.7	C124—C123—C122	118.9 (4)
C31—C30—H30	118.7	C124—C123—H123	120.5
C32—C31—C30	117.7 (4)	C122—C123—H123	120.5
C32—C31—H31	121.1	C123—C124—C125	119.6 (3)
C30—C31—H31	121.1	C123—C124—H124	120.2
C31—C32—C33	121.0 (4)	C125—C124—H124	120.2
C31—C32—H32	119.5	N17—C125—C124	122.2 (3)
C33—C32—H32	119.5	N17—C125—C126	116.2 (3)
C32—C33—C34	118.8 (4)	C124—C125—C126	121.5 (3)
C32—C33—H33	120.6	N18—C126—C127	122.4 (3)
C34—C33—H33	120.6	N18—C126—C125	116.4 (3)
N5—C34—C33	121.3 (4)	C127—C126—C125	121.2 (3)
N5—C34—C35	115.8 (3)	C128—C127—C126	118.8 (3)
C33—C34—C35	122.9 (3)	C128—C127—H127	120.6
N6—C35—C36	121.1 (3)	C126—C127—H127	120.6
N6—C35—C34	116.0 (3)	C129—C128—C127	119.2 (4)
C36—C35—C34	122.9 (3)	C129—C128—H128	120.4
C37—C36—C35	118.8 (4)	C127—C128—H128	120.4
C37—C36—H36	120.6	C128—C129—C130	117.8 (4)
C35—C36—H36	120.6	C128—C129—H129	121.1
C38—C37—C36	120.1 (3)	C130—C129—H129	121.1
C38—C37—H37	120.0	N18—C130—C129	124.4 (4)
C36—C37—H37	120.0	N18—C130—H130	117.8
C37—C38—C39	118.6 (4)	C129—C130—H130	117.8
C37—C38—H38	120.7	N19—C131—C132	124.1 (3)
C39—C38—H38	120.7	N19—C131—H131	118.0
N6—C39—C38	122.1 (4)	C132—C131—H131	118.0
N6—C39—H39	118.9	C133—C132—C131	118.1 (3)
C38—C39—H39	118.9	C133—C132—H132	120.9
N7—C40—C41	122.1 (3)	C131—C132—H132	120.9
N7—C40—H40	118.9	C132—C133—C134	119.0 (3)
C41—C40—H40	118.9	C132—C133—H133	120.5
C40—C41—C42	119.1 (3)	C134—C133—H133	120.5
C40—C41—H41	120.4	C133—C134—C135	119.1 (3)
C42—C41—H41	120.4	C133—C134—H134	120.4
C43—C42—C41	119.1 (3)	C135—C134—H134	120.4
C43—C42—H42	120.4	N19—C135—C134	122.4 (3)
C41—C42—H42	120.4	N19—C135—C136	116.7 (3)
C42—C43—C44	119.0 (3)	C134—C135—C136	120.9 (3)
C42—C43—H43	120.5	N20—C136—C137	121.8 (3)
C44—C43—H43	120.5	N20—C136—C135	116.7 (3)
N7—C44—C43	121.2 (3)	C137—C136—C135	121.5 (3)
N7—C44—C45	114.7 (3)	C138—C137—C136	119.8 (3)
C43—C44—C45	124.1 (3)	C138—C137—H137	120.1
N8—C45—C46	121.8 (3)	C136—C137—H137	120.1
N8—C45—C44	114.9 (3)	C137—C138—C139	118.6 (3)
C46—C45—C44	123.2 (3)	C137—C138—H138	120.7
C45—C46—C47	118.7 (3)	C139—C138—H138	120.7
C45—C46—H46	120.7	C140—C139—C138	118.6 (3)
C47—C46—H46	120.7	C140—C139—H139	120.7
C48—C47—C46	119.5 (3)	C138—C139—H139	120.7
C48—C47—H47	120.3	N20—C140—C139	123.7 (3)
C46—C47—H47	120.3	N20—C140—H140	118.1
C47—C48—C49	118.3 (3)	C139—C140—H140	118.1
C47—C48—H48	120.8	H74V—O74W—H74W	114 (5)
C49—C48—H48	120.8	H76W—O76W—H76V	114 (6)
N8—C49—C48	123.5 (3)	H77W—O77W—H77V	83 (5)
N8—C49—H49	118.2	H78W—O78W—H78V	121 (6)
C48—C49—H49	118.2	H79W—O79W—H79V	119 (4)
C56—C51—C52	119.5 (3)	H80V—O80W—H80W	116 (5)
C56—C51—C57	119.7 (3)	H81W—O81W—H81V	102 (4)
C52—C51—C57	120.7 (3)	O83W—O83V—O96W	172.9 (12)
C53—C52—C51	120.3 (3)	H84W—O84W—H84V	72 (4)
C53—C52—H52	119.9	H85W—O85W—H85V	99 (4)
C51—C52—H52	119.9	H86W—O86W—H86V	103 (4)
C52—C53—C54	119.5 (3)	H87V—O87W—H87W	89 (4)
C52—C53—C58	121.1 (3)	H88W—O88W—H88V	108 (5)
C54—C53—C58	119.2 (3)	H89V—O89W—H89W	119 (4)
C55—C54—C53	120.4 (3)	H90W—O90W—H90V	99 (4)
C55—C54—H54	119.8	H91V—O91W—H91W	101 (4)
C53—C54—H54	119.8	H92W—O92W—H92V	110 (4)
C54—C55—C56	119.9 (3)	H93V—O93W—H93W	104 (4)
C54—C55—C59	119.6 (3)	H94W—O94W—H94V	65 (3)
C56—C55—C59	120.6 (3)	H95W—O95W—H95V	100 (4)
C51—C56—C55	120.4 (3)	O83V—O96W—H96W	121 (4)
C51—C56—H56	119.8	H97V—O97W—H97W	104 (4)
C55—C56—H56	119.8	H98W—O98W—H98V	102 (4)
O8—C57—O7	124.0 (3)	H99W—O99W—H99V	132 (4)

### Hydrogen-bond geometry (Å, °)

**Table:**

*D*—H···*A*	*D*—H	H···*A*	*D*···*A*	*D*—H···*A*
O5—H5W···O85W	0.87 (4)	1.72 (2)	2.565 (3)	165 (4)
O85W—H85V···O97W	0.847 (19)	1.87 (2)	2.698 (4)	164 (4)
O85W—H85W···O93W	0.85 (4)	1.86 (2)	2.707 (4)	175 (4)
O93W—H93V···O16	0.843 (19)	1.92 (2)	2.745 (3)	166 (5)
O93W—H93W···O87W	0.84 (4)	1.88 (2)	2.710 (4)	176 (5)
O97W—H97V···O16	0.86 (4)	1.88 (2)	2.735 (3)	171 (4)
O97W—H97W···O74W	0.879 (19)	1.87 (2)	2.733 (4)	167 (4)
O87W—H87W···O6	0.838 (19)	2.03 (2)	2.868 (4)	177 (5)
O87W—H87V···O13	0.85 (4)	1.89 (2)	2.734 (4)	174 (5)
O74W—H74V···O91W	0.83 (2)	1.96 (2)	2.776 (4)	170 (5)
O74W—H74W···O83W	0.845 (19)	1.95 (3)	2.696 (5)	146 (5)
O91W—H91V···O80W	0.863 (19)	1.89 (2)	2.750 (4)	176 (5)
O91W—H91W···O79W	0.87 (4)	2.03 (2)	2.882 (4)	169 (4)
O80W—H80V···O15	0.83 (4)	2.03 (3)	2.796 (3)	154 (5)
O80W—H80W···O81W^i^	0.885 (19)	1.92 (2)	2.788 (4)	167 (4)
O98W—H98V···O15	0.819 (19)	1.97 (2)	2.789 (3)	176 (4)
O98W—H98W···O81W^i^	0.823 (19)	2.00 (3)	2.753 (4)	152 (4)
O81W—H81W···O12	0.85 (4)	1.98 (2)	2.820 (4)	170 (5)
O81W—H81V···O92W	0.825 (19)	1.88 (2)	2.692 (4)	169 (5)
O92W—H92W···O23	0.862 (19)	1.91 (2)	2.757 (4)	169 (4)
O92W—H92V···O99W	0.829 (19)	2.01 (2)	2.801 (4)	159 (4)
O99W—H99W···O85W	0.847 (19)	1.93 (2)	2.752 (4)	164 (4)
O99W—H99V···O96W	0.851 (19)	2.06 (3)	2.845 (5)	153 (4)
O96W—H96W···O24	0.96 (5)	1.75 (4)	2.533 (6)	137 (3)
O22—H22V···O14^ii^	0.86 (2)	1.67 (2)	2.518 (3)	167 (5)

Symmetry codes: (i) *x*+1, *y*, *z*; (ii) *x*, *y*+1, *z*.
